# Calcium Competitive Inhibition of Langerin by Thiazolopyrimidinones

**DOI:** 10.1021/acs.jmedchem.5c01756

**Published:** 2025-11-19

**Authors:** Yunzhan Ning, Nina-Louisa Efrém, Machoud Amoussa, Ertan Turhan, Dazhong Zheng, Jonathan Lefèbre, Max Ruwolt, Ursula Neu, Maurice Besch, Bernhard Loll, Dennis Kurzbach, Jesko Köhnke, Marc Nazaré, Christoph Rademacher

**Affiliations:** Department of Pharmaceutical Sciences and Vienna Doctoral School of Pharmaceutical, Nutritional and Sport Sciences, University of Vienna, Vienna 1090, Austria; Department of Chemical Biology, Leibniz-Forschungsinstitut für Molekulare Pharmakologie (FMP), Berlin 13125, Germany; Institute of Biological Chemistry, Faculty of Chemistry, University of Vienna, Vienna 1090, Austria; University of Vienna, Vienna 1090, Austria; Institute of Food Chemistry, Leibniz University Hannover, Hannover 30167, Germany; School of Chemistry, University of Glasgow, Glasgow G12 8QQ, United Kingdom; Department of Pharmaceutical Sciences and Vienna Doctoral School of Pharmaceutical, Nutritional and Sport Sciences, University of Vienna, Vienna 1090, Austria; Institute of Chemistry and Biochemistry, Laboratory of Structural Biochemistry, Freie Universität Berlin, Berlin 14195, Germany; Department of Pharmaceutical Sciences and Vienna Doctoral School of Pharmaceutical, Nutritional and Sport Sciences, University of Vienna, Vienna 1090, Austria; Institute of Chemistry and Biochemistry, Laboratory of Structural Biochemistry, Freie Universität Berlin, Berlin 14195, Germany; Institute of Biological Chemistry, Faculty of Chemistry, University of Vienna, Vienna 1090, Austria; University of Vienna, Vienna 1090, Austria; Institute of Food Chemistry, Leibniz University Hannover, Hannover 30167, Germany; School of Chemistry, University of Glasgow, Glasgow G12 8QQ, United Kingdom; Department of Chemical Biology, Leibniz-Forschungsinstitut für Molekulare Pharmakologie (FMP), Berlin 13125, Germany; Department of Pharmaceutical Sciences and Vienna Doctoral School of Pharmaceutical, Nutritional and Sport Sciences, University of Vienna, Vienna 1090, Austria, Max Perutz Laboratories, Vienna Biocenter Campus (VBC), Vienna 1030, Austria

## Abstract

C-Type lectins are a large family of carbohydrate-binding proteins. Langerin is a member of this family and is expressed by Langerhans cells, involved in pathogen recognition and innate immune activation, making it a target for small-molecule modulation in immunology and infectious diseases. We previously identified thiazolopyr-imidinones as a series of allosteric inhibitors, but the underlying mechanism remained unclear. In this study, ^43^Ca NMR demonstrated that these fragments induce Ca^2+^ release from the receptor. Our ITC data suggested a competitive relationship between inhibitors and Ca^2+^, which was further validated by ^19^F NMR spectroscopy showing inhibition of carbohydrate binding. Surprisingly, the fragment binding site was found to be located beneath the long loop, which supports the dynamic nature of the long loop being highly Ca^2+^ dependent. Our findings provide insight into the novel Ca^2+^-competitive inhibitory mechanism of murine langerin and are the first report on such an inhibitory mechanism for a C-type lectin.

**Figure:**
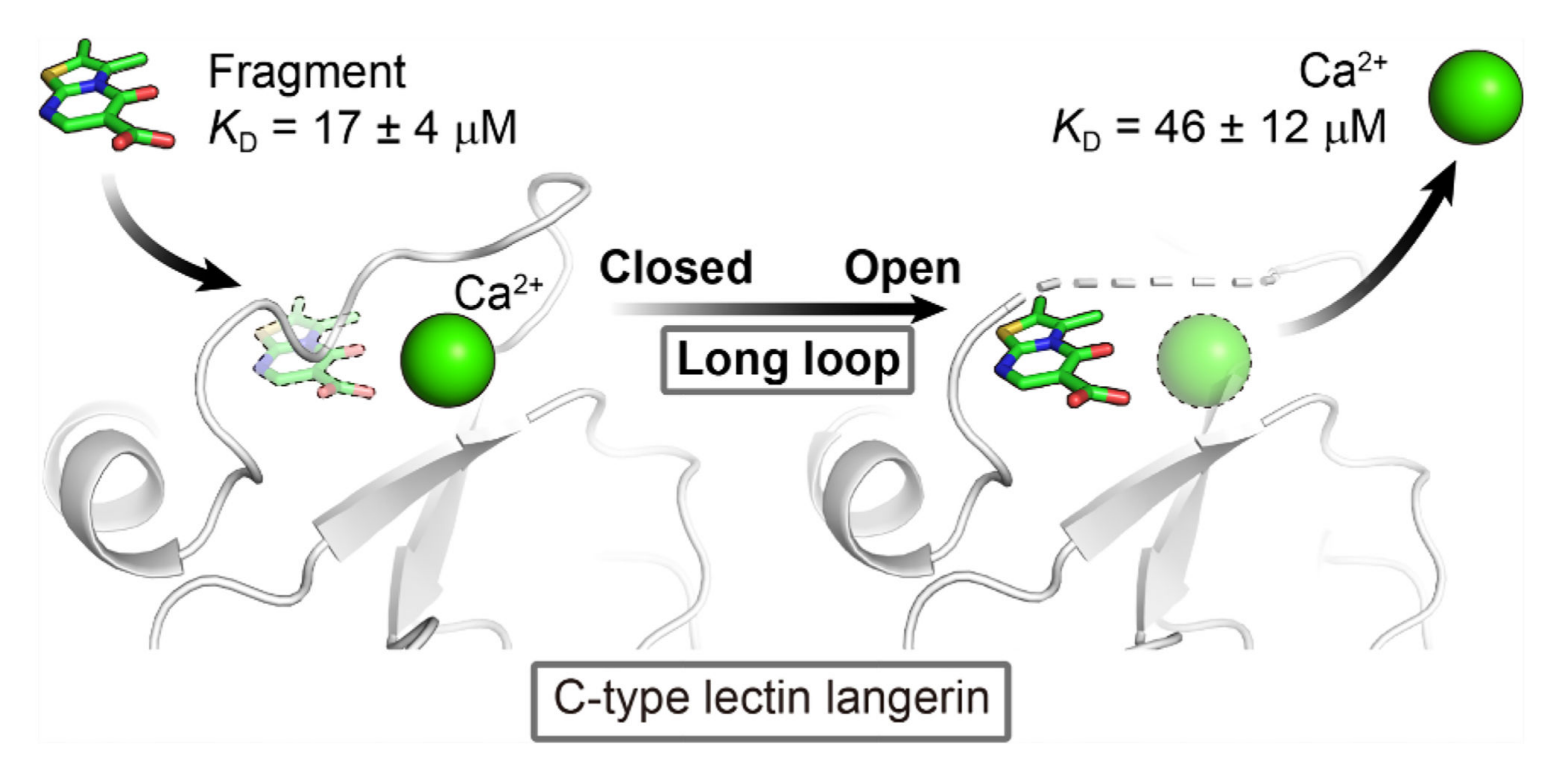

## Introduction

Langerin, a member of the C-type lectin (CTL) superfamily, is a Ca^2+^-dependent carbohydrate-binding protein. This receptor is primarily expressed on Langerhans cells, antigen-presenting cells residing in the epidermis of the skin, where it plays a crucial role in various biological processes, in particular, in initiating the innate immune response. It is involved in pathogen recognition, immune signaling, and modulation of immune responses.^1–10^ Notably, langerin is involved in HIV recognition and internalization, a process that heavily relies on the formation of the Birbeck granules, which are specialized organelles unique to Langerhans cells.^11–15^ Langerin’s involvement in immune responses makes it a promising target for inhibitor development, enhancing our understanding of its biological role and revealing its therapeutic potential.

Various ligands for langerin have been reported, including glycomimetic compounds,^16–19^ antibodies,^20–24^ and allosteric noncarbohydrate inhibitors.^25^ However, the exploration of drug-like heterocyclic inhibitors is challenging due to the low druggability of langerin. This is a problem inherent to many glycan-binding proteins for their hydrophilic and solvent-exposed carbohydrate-binding sites.^26,27^ Fragment-based drug discovery offers a promising avenue for addressing this challenge by identifying small molecules that bind to secondary sites, which can then be optimized to become more potent drug candidates. The canonical carbohydrate-binding site of CTLs features a long loop where the EPN (Glu-Pro-Asn) motif, responsible for its mannose specificity, is located. The central proline in the EPN motif is crucial for the Ca^2+^ coordination. In the *holo* (Ca^2+^-bound) state, this proline adopts a *cis* conformation, positioning the long loop to form the Ca^2+^ cage characteristic for all Ca^2+^-binding CTL-like domains.^28,29^

The recognition of the cofactor Ca^2+^ by langerin is tightly coupled to its life cycle as an endocytic and recycling receptor.^30^ Being exposed on the surface of Langerhans cells, langerin interacts with carbohydrate ligands presented on pathogens mediated by Ca^2+^. Following this initial binding event, the langerin-pathogen complex is endocytosed, and langerin releases its cargo into the endosomal lumen.^31^ Similar to other CTLs, this release is likely driven by two factors.^32^ First, the Ca^2+^ affinity of langerin drops due to the pH decrease from around pH 7 in the extracellular medium to around pH 6 in the early endosome.^33^ Second, a significant reduction of the Ca^2+^ concentration, from millimolar in the extracellular space to lower micromolar in the early endosomes.^34^ Previously, we and others have described the interplay between Ca^2+^ affinity and pH for langerin and other CTLs, where the long loop plays an important role.^31,33,35,36^ These studies imply that conformational changes in the long loop are likely to be of considerable biological significance.

Ca^2+^ binding to langerin affects the arrangement of loop structures, which in turn influences the recognition of a previously identified thiazolopyrimidinone-based allosteric inhibitor with two-digit micromolar affinity.^25^ Our nuclear magnetic resonance (NMR) and surface plasmon resonance (SPR) data suggested that Ca^2+^ binding was unaffected by these inhibitors and, instead, Ca^2+^ recognition induced the formation of the inhibitor binding site. Additionally, a recent study localized the binding site in the cleft between the short and long loops opposite to the canonical carbohydrate binding site.^37^ Supporting this notion, X-ray crystallographic structures of langerin showed a tryptophan binding in the same cleft, reinforcing this region as a potential small molecule binding site (PDB ID: 5G6U and 3P7H).^38,39^ These findings prompted us to investigate the exact inhibitor binding site of the original thiazolopyrimidinone fragments on the murine langerin carbohydrate recognition domain (CRD, amino acids: 191−331) and how this site is shaped in the presence of Ca^2+^.

## Results and Discussion

### Thiazolopyrimidinones Inhibit Ca^2+^ Binding to Murine Langerin

**Figure 1:**
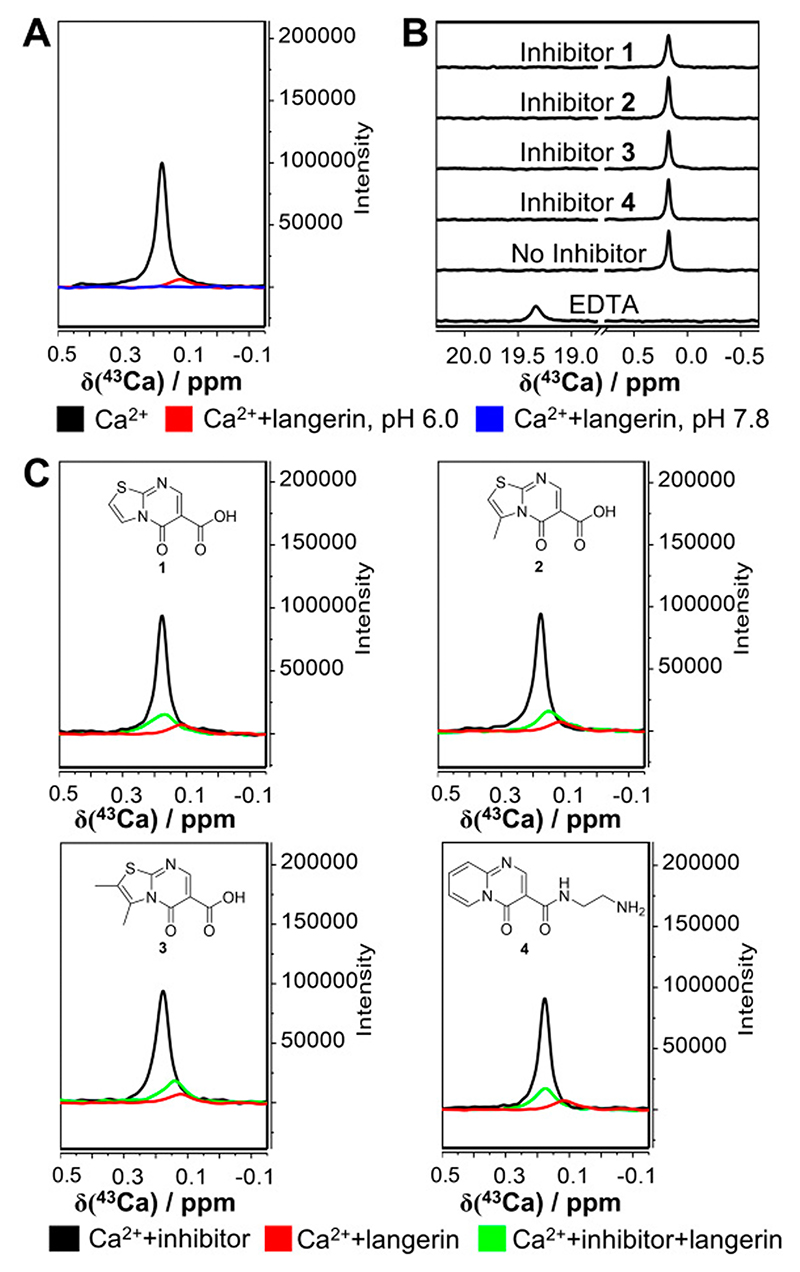
^43^Ca NMR assay suggests 1−4 inhibit Ca^2+^ binding to murine langerin. (A) Superposed spectra of 2.5 mM ^43^CaCl_2_ under various conditions: 25 mM MES/NaOH, 40 mM NaCl, pH 6.0 (control, black); with 50 *μ*M langerin CRD in 25 mM MES/NaOH, 40 mM NaCl, pH 6.0 (red); with 50 *μ*M langerin CRD in 50 mM Tris/HCl, 150 mM NaCl, pH 7.8 (blue). The chemical shift perturbation and signal reduction illustrate pH-dependent binding of Ca^2+^ to langerin. (B) Controls to exclude the direct interaction of Ca^2+^ and inhibitors. Positive control: 2.5 mM ^43^CaCl_2_ with 5 mM EDTA, showing complete chelation of Ca^2+^. Negative controls: 2.5 mM ^43^CaCl_2_ with 400 *μ*M inhibitors **1−4**. No interactions are observed between Ca^2+^ and inhibitors in the absence of langerin. (C) ^43^Ca NMR spectra comparing the effect of 400 *μ*M inhibitors (**1−4**) on Ca^2+^ binding to langerin under 2.5 mM ^43^CaCl_2_ in 25 mM MES/NaOH, 40 mM NaCl, pH 6.0. Spectra of Ca^2+^ in absence (black) and in the presence of 50 *μ*M langerin CRD (red) served as controls. Significant chemical shifts toward the unbound state and increased signal intensity in the langerin-inhibitor complexes (green) indicate competitive inhibition of Ca^2+^ binding by the inhibitors.

The presence of thiazolopyrimidinone fragments decreases the affinity of murine langerin (amino acids: 191−331) toward carbohydrates but not for Ca^2+^, an observation that warranted further investigation.^25^ To this end, we first investigated the Ca^2+^ recognition of langerin employing ^43^Ca NMR spectroscopy.^40–46^ In the spectrum of a 2.5 mM isotope-enriched calcium chloride solution, a sharp peak appeared at 0.176 ppm, representing the free state of Ca^2+^ (Figure 1A). Upon addition of 50 *μ*M langerin, a reduced single signal with significant line broadening was observed at 0.116 ppm (Table S1), indicating binding of the cofactor to the receptor, which is notable given the 50-fold excess of Ca^2+^ over protein and hence the much lower ratio of Ca^2+^ in the bound state compared to the free state. This effect was also observed for the Ca^2+^-EDTA complex, which served as a positive control (Figure 1B). At pH 7.8 (Figure 1A and Figure S2), the line broadening and signal reduction were further enhanced compared to pH 6.0, suggesting stronger complex formation at higher pH.

We then selected the smallest fragments from our previous screening campaign,^25^ driven by the observation that tryptophan bound between the two loops shares a similar polar surface area with thiazolopyrimidinones, suggesting a shared binding site (PDB ID: 5G6U and 3P7H).^38,39^ We chose structures (**1, 2, 3**) with previously reported affinities of 0.7, 1.2, and 0.9 mM in the presence of Ca^2+^.^25^ Compound **4** was included as the isostere of inhibitor **1**. An (ethyl)amine linker was introduced due to the poor solubility of the carboxylic acid derivative (Figure S1).

Next, we aimed to confirm that Ca^2+^ binding is unaffected by inhibitor binding using the ^43^Ca NMR assay. However, the results showed shifted Ca^2+^ signals toward the free state (*δ* = 0.168, 0.149, 0.138, and 0.175 ppm for inhibitors **1−4**, respectively, Figure 1C and Figure S3) and increased signal intensity compared to the signal in the presence of receptor only (Table S1), indicating partial release of Ca^2+^ from langerin upon inhibitor binding at pH 6. This was supported by similar observations at pH 7.8, although the lower signal-to-noise ratio rendered a more detailed analysis difficult (Figure S2). Due to the limited solubility of the compounds and the need to maintain sufficient signal-to-noise for reliable ^43^Ca signal detection, a high concentration of Ca^2+^ was required, precluding the use of equimolar concentrations of Ca^2+^ and inhibitor. We ruled out direct Ca^2+^ complexation with the inhibitors by performing experiments in the absence of the receptor (Figure 1B). In contrast to our previous results using more elaborate structures from this series, where *N*-(2-(2,3-dihydroimidazo[2,1-*b*]-thiazol-6-yl)-ethyl)-5-oxo-5*H*-thiazolo-[3,2-*a*]-pyrimidine-6-carboxamide did not show competition with Ca^2+^, these findings suggested that Ca^2+^ is partially released from the receptor upon binding of the fragments **1−4**.^25^

### Inhibition of Ca^2+^ Binding to Murine Langerin Is Achieved through Competition

**Figure 2:**
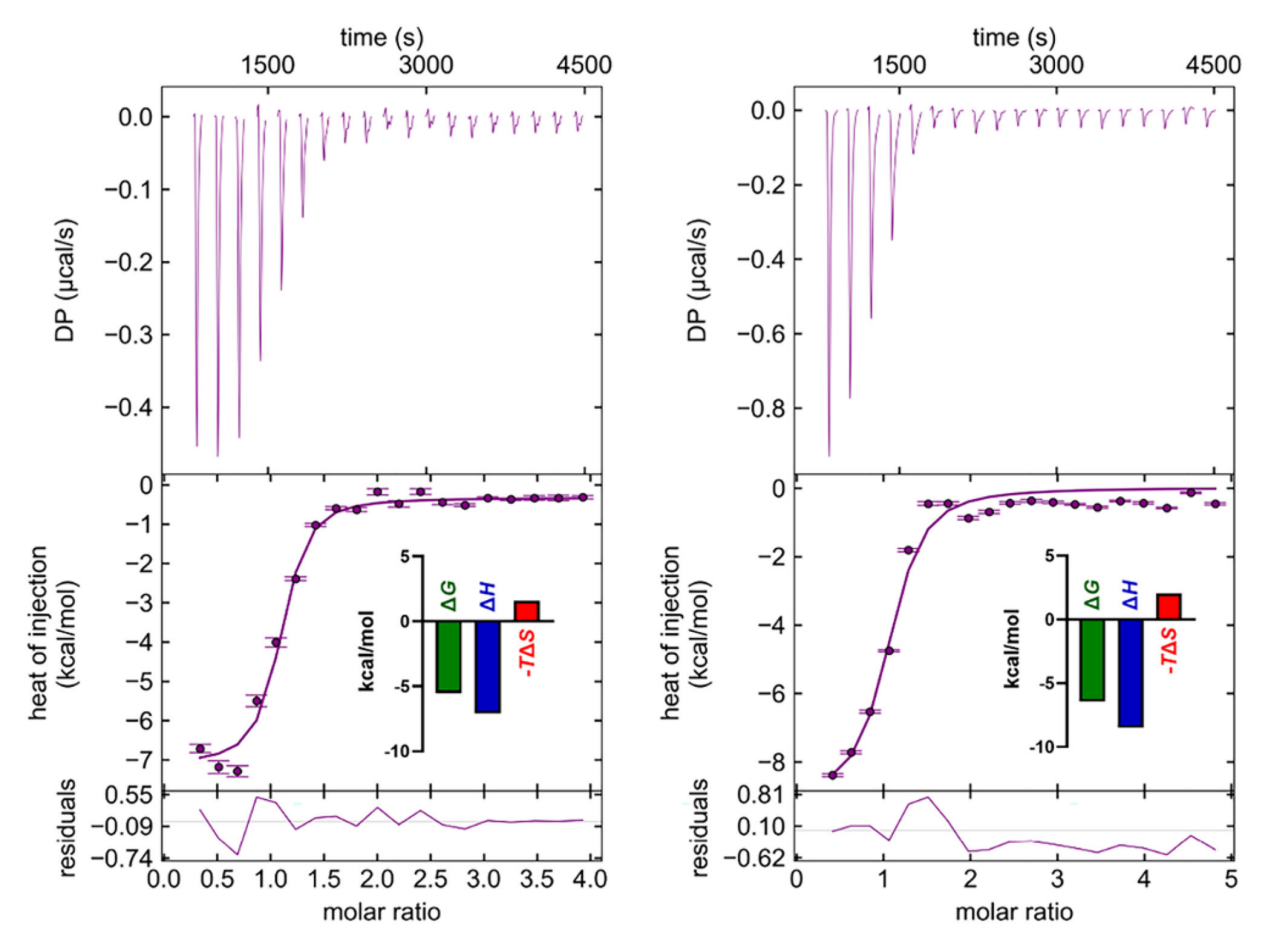
Representative thermograms and binding isotherm from ITC experiments measuring binding of Ca^2+^ to murine langerin CRD at different pH units. Thermogram and binding isotherm of Ca^2+^ at pH 6.0, yielding *K*_D_ = 46 ± 12 *μ*M, Δ*H* = −7.1 ± 1.9 kcal/mol, and −*T*Δ*S*= 1.6 ± 0.4 kcal/mol (left panel). Ca^2+^ binding data at pH 7.8, with *K*_D_ = 8.5 ± 2.8 *μ*M, Δ*H* = −8.5 ± 2.2 kcal/mol, and −*T*Δ*S*= 2.0 ± 0.5 kcal/mol, reflecting an enhanced Ca^2+^ affinity under slightly basic conditions (right panel). The differences in binding affinity highlight the pH dependency of Ca^2+^-langerin interactions, consistent with the functional role of langerin in various physiological environments.

To further investigate this inhibition of Ca^2+^ binding, we first measured Ca^2+^ affinity toward murine langerin using isothermal titration calorimetry (ITC). While other CTLs can harbor multiple Ca^2+^ binding sites, langerin contains a single site, allowing for straightforward fitting using a single-site model.^9^ The measured affinities are more than 10-fold higher than those reported for the human homolog, although both proteins exhibit similar pH dependence, with affinity decreasing at a lower endosomal pH level (Figure 2). Moreover, this affinity difference aligns with the increased line broadening and lower signal-to-noise ratio observed in ^43^Ca NMR at pH 6.0 compared to data collected at pH 7.8 (Figure 1A and Figure S2). However, these ITC-derived Ca^2+^ affinities differ from those obtained via ^1^H−^15^N-HSQC NMR titrations,^25^ likely due to the threshold protein concentration required in NMR, which can limit accuracy in high-affinity measurements. Notably, the high affinity measured by ITC also explains why previous NMR experiments showed no apparent change in the Ca^2+^ affinity in the presence of the allosteric inhibitor.

**Figure 3:**
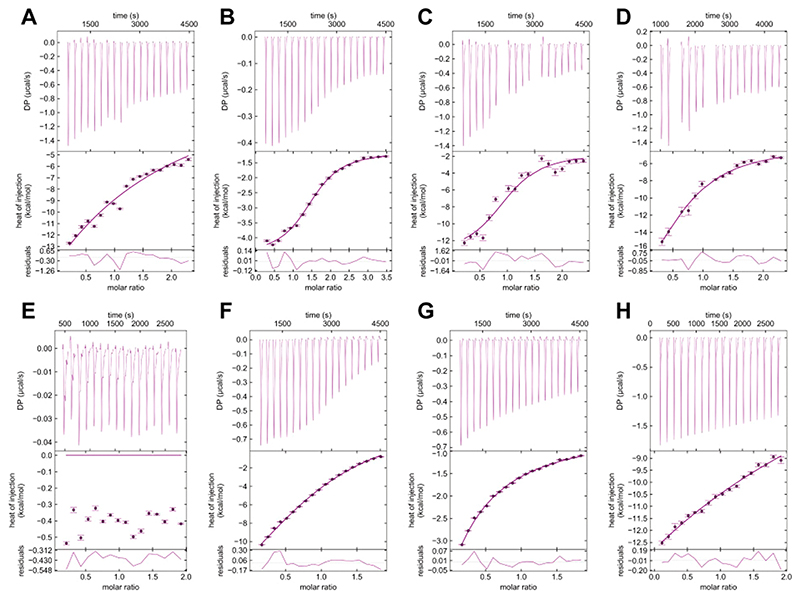
ITC thermograms and binding isotherms demonstrating Ca^2+^-competitive binding of inhibitors 1−4 to murine langerin. (A−D) Thermograms and binding isotherms for inhibitors **1−4** titrated langerin in the absence of Ca^2+^. (E−H) Thermograms and binding isotherms for inhibitors **1−4** titrated into langerin in the presence of Ca^2+^. Due to the solubility limit from inhibitors, saturation could not be achieved in the presence of Ca^2+^. All raw data are processed using NITPIC,^47^ fitted using SEDPHAT,^48^ and presented using GUSSI.^49^ Data artifacts and statistical outliers were excluded from the final fits.

Using the ITC-based Ca^2+^ affinities as a reference, we conducted titrations of inhibitors **1−4** in the absence and presence of Ca^2+^ (Figure 3). In the absence of Ca^2+^, inhibitor titrations (Figure 3A−D) yielded conclusive heat responses. Fitting these data to a single-site model, **1** and **4** showed hyperbolic curves and gave *K*_D_ = 1.1 ± 0.3 mM and *K*_D_ = 200 ± 90 *μ*M, respectively (Figure 3A,D, Table 1). Inhibitors 2 and 3, in contrast, showed sigmoidal curves with *K*_D_ = 19 ± **2** and 53 ± 8 *μ*M, respectively (Figure 3B,C, Table 1). In the presence of 5 mM Ca^2+^, 1 exhibited a weak heat response, suggesting minimal binding, which might be obscured by its weaker affinity and minor enthalpy relative to that of Ca^2+^ (Figure 3E). The heat integration for inhibitors **2−4** in the presence of Ca^2+^ showed hyperbolic curves. When fitted to a single-site model, the affinity data deviated strongly from the data collected in the absence of Ca^2+^ (Figure S4, Table 1). Taking our ^43^Ca NMR data into consideration, we applied a competitive binding model that assumes competition between inhibitors **2−4** and Ca^2+^. This model provided better fits compared to the single-site model (Figure 3F−H, Figure S4), yielding affinities consistent with those obtained from measurements in the presence of Ca^2+^ (Table 1). For example, inhibitors **3** and **4** showed 17 ± 4 and 480 ± 70 *μ*M affinity, which were comparable to 53 ± 8 and 200 ± 90 *μ*M from conditions in the absence of Ca^2+^. These results further supported the competitive inhibition mechanism.

Ligand efficiency (LE) calculations based on the best affinities from both conditions indicated moderate values for inhibitors **1** and **4** (0.31 and 0.27 kcal·mol^−1^·HA^−1^) and high efficiencies for inhibitors **2** and **3** (0.46 and 0.44 kcal·mol^−1^· HA^−1^). This suggests that the methyl substituents on the thiazolopyrimidinone core likely influence the binding mode and overall affinity. These significant differences in LE between similar structures warrant further investigation of the binding site and exploration by an array of closely similar substituted analogues.

### Competition with Ca^2+^ Inhibits Carbohydrate Binding to Murine Langerin

**Figure 4:**
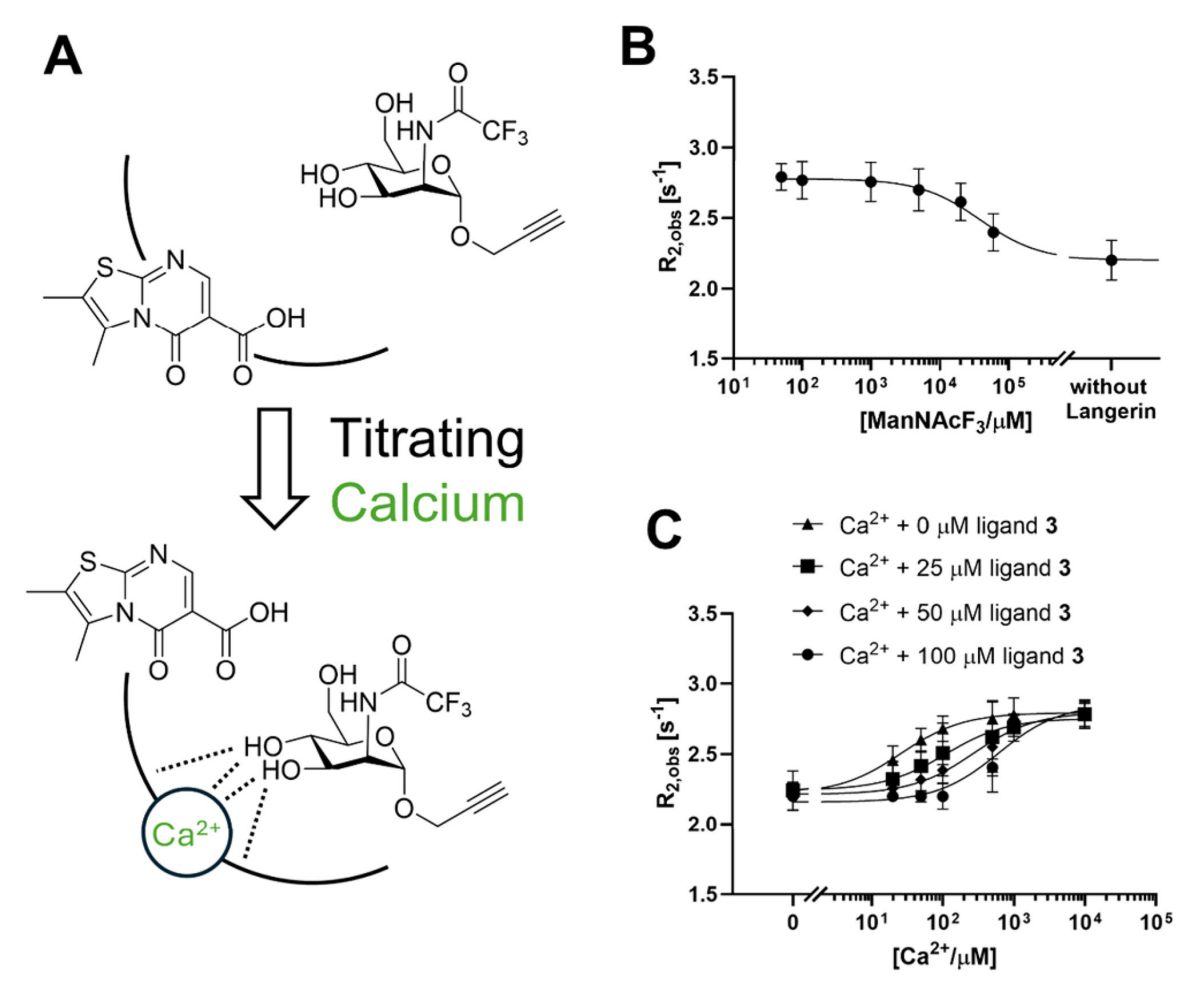
^19^F R_2_-filtered NMR reveals the inhibition of carbohydrate binding to murine langerin. (A) Schematic representation of the assay. In the absence of Ca^2+^, inhibitor **3** occupies the binding site, blocking carbohydrate reporter ManNAcF_3_ from binding. Adding Ca^2+^ competes with the inhibitor **3**, enabling Ca^2+^ coordination and the carbohydrate reporter to bind. (B) Titration of the carbohydrate reporter into langerin CRD yielded a *K*_D_ of 28 ± 5 mM at 25 mM MES/NaOH, 40 mM NaCl, and 5 mM CaCl_2_, pH 6.0, with *R*_2,max_ = 2.79 s^−1^ and *R*_2,f_ = 2.20 s^−1^. (C) Competition assay with 50 *μ*M reporter and 50 *μ*M protein. The EC_50_ of the carbohydrate reporter shifted from 26 to 610 *μ*M upon increasing the inhibitor **3** concentration from 0 to 100 *μ*M, supporting the Ca^2+^-competitive inhibition mechanism.

Based on these findings, we concluded that **1−4** compete with Ca^2+^. To validate this relationship, we explored whether this competition translates into inhibition of carbohydrate binding using a previously developed mannoside reporter ManNAcF_3_.^51^ The reporter binds to langerin in a Ca^2+^-dependent manner, and the binding can be quantitatively analyzed using ^19^F NMR. A calcium chloride solution was titrated into the sample comprising the receptor and mannoside reporter in the presence of various concentrations of the most potent inhibitor **3** (Figure 4A). In the absence of **3**, the addition of Ca^2+^ yielded an EC_50_ = 26 ± 5 *μ*M, which is consistent with the Ca^2+^ affinity determined by ITC (Figure 2). Increasing concentrations of **3** resulted in increased EC_50_ values, indicating the inhibition of carbohydrate reporter binding (Figure 4C). These results demonstrated the inhibition of carbohydrate binding through Ca^2+^ competition by inhibitor **3**.

### X-ray Crystallography Reveals Binding Site for 3

**Figure 5:**
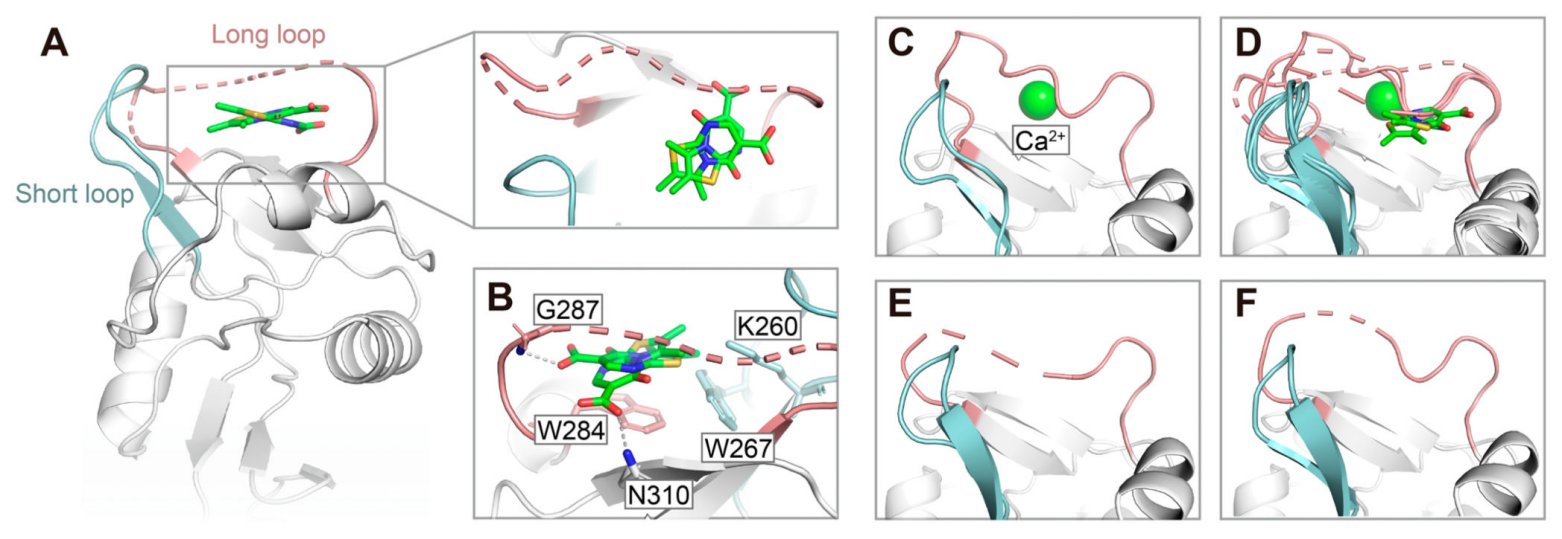
Binding site of inhibitor 3 and Ca^2+^-dependent conformational dynamics of the long loop in murine langerin (PDB ID: 9RKO and 9HYE). (A) Crystal structure of murine langerin CRD in complex with inhibitor **3** reveals a flexible, open conformation of the long loop and the absence of Ca^2+^ (PDB ID: 9RKO). The close-up view highlights two potential binding modes of **3**, with the carboxylic acid oriented differently in each pose. The dashed line indicates missing electron density due to loop flexibility. (B) Key residues forming the binding pocket of **3** include Trp284, Trp267, and Lys260, with hydrogen bonding to the N*α* of Gly287, with a distance of 3.2 Å and charge-assisted hydrogen bond formation with the side-chain amide of Asn310. (C) Ca^2+^-bound structure of murine langerin (PDB ID: 9RKO) showing a well-ordered long loop, closely matching the previously reported Ca^2+^-bound state (PDB ID: 5M62).^54^ (D) Superposition of the four chains from both structures highlights marked differences in the long loop conformation, with only minor variability in the short loop. (E, F) The structure of murine langerin in the absence of Ca^2+^ (PDB ID: 9HYE) shows two chains with disordered long loops and *cis* conformation of Pro289, reinforcing Ca^2+^-sensitive loop mobility. The long loop is shown in light red, short loop in light blue, inhibitor **3** as a green stick, and Ca^2+^ as a green sphere.

To further elucidate the structural basis of Ca^2+^ competition, we conducted X-ray crystallographic studies. **3** was cocrystallized in complex with murine langerin CRD in the presence of Ca^2+^, and the resulting protein crystals yielded a structure at 1.89 Å resolution (PDB ID: 9RKO, Table S2). The asymmetric unit contained two protomers, with one protomer bound to **3**, while the other bound to Ca^2+^. The inhibitor-bound protomer is Ca^2+^-free and exhibits an open long loop with significant mobility, creating a pocket that accommodates 3 (Figure 5A). This agrees with our previous finding that the thiazolopyr-imidinone binding site is located in the cleft between the long loop and short loop.^37^ The pocket is constrained, further supporting the hypothesis that the methyl group at position 3 of the thiazolopyrimidinone, which is the only structural difference between compounds **2** and **3**, increases the shape complementarity in the constrained binding pocket. Careful refinement of inhibitor occupancy and orientation revealed a superposition of two closely related binding modes with a roughly 1:1 occupancy: one engages in hydrogen bonding with the N*α* of Gly287, and the other forms a hydrogen bond with the side-chain amide of Asn310 (Figure 5B and Figure S5). This is further supported by docking studies, which revealed different orientations of **3** within the binding pocket, highlighting the dynamic nature of the interaction (Figure S6). Owing to its small size and minimal polar functionality, the binding of fragment **3** appears to be primarily driven by van der Waals forces, ***π−π*** stacking with Trp284 and hydrophobic contacts with Trp267. In contrast, the Ca^2+^-bound chain displays a well-defined long loop conformation and strongly resembles the previously reported Ca^2+^-bound structure (PDB ID: 5M62) with a backbone RMSD of 0.291 Å for 139 pairs of C_*α*_ atoms (Figure 5C).

A structure of murine langerin in the absence of Ca^2+^ was also solved (PDB ID: 9HYE) and reinforces the dynamic nature of the long loop. In this structure, Pro289 remains in the *cis* conformation while the long loop shows significant mobility, as indicated by the missing electron density, similar to the inhibitor-bound structure (Figure 5D−F). This observation is consistent with previous NMR results showing 75 ± 10% of the central proline adopts the *cis* conformation in Ca^2+^-free human langerin.^33^ The pronounced long loop conformational differences between Ca^2+^-bound and unbound states highlight the sensitivity of the long loop to Ca^2+^. A similar mechanism has been described for another CTL, DC-SIGNR,^52^ where the loop adjacent to its secondary Ca^2+^ site shifts between open and closed states depending on Ca^2+^ binding. Therefore, it remains unclear whether the long loop structural rearrangement upon inhibitor binding is driven either by an induced fit mechanism, conformational selection, or a combination of both.^53^

These findings support the hypothesis that fragments **1−4** inhibit binding of Ca^2+^ to langerin through direct competition and that their binding pocket is located near the Ca^2+^ binding site under the long loop.

### Potential Secondary Site for Thiazolopyrimidinone Inhibitors

Attempts to solve the structures of **1**, **2**, and **4** in complex with murine langerin using X-ray crystallography were unsuccessful. Therefore, we employed solvent paramagnetic relaxation enhancement (sPRE) NMR. This technique employs soluble paramagnetic probes to assess the solvent accessibility of the protein backbone amides via ^1^H−^15^N HMQC NMR.^55^ Backbone chemical shifts of murine langerin have been reported,^37^ but resonances in the dynamic long loop were mostly unassigned, limiting information from that region. The resulting ΔsPRE values from inhibitor binding in the absence and presence of 5 mM Ca^2+^ were mapped onto the Ca^2+^-bound X-ray structure (PDB ID: 5M62), with positive signals denoting lower probe accessibility around the backbone and negative signals suggesting higher probe accessibility around the backbone during ligand binding.

**Figure 6:**
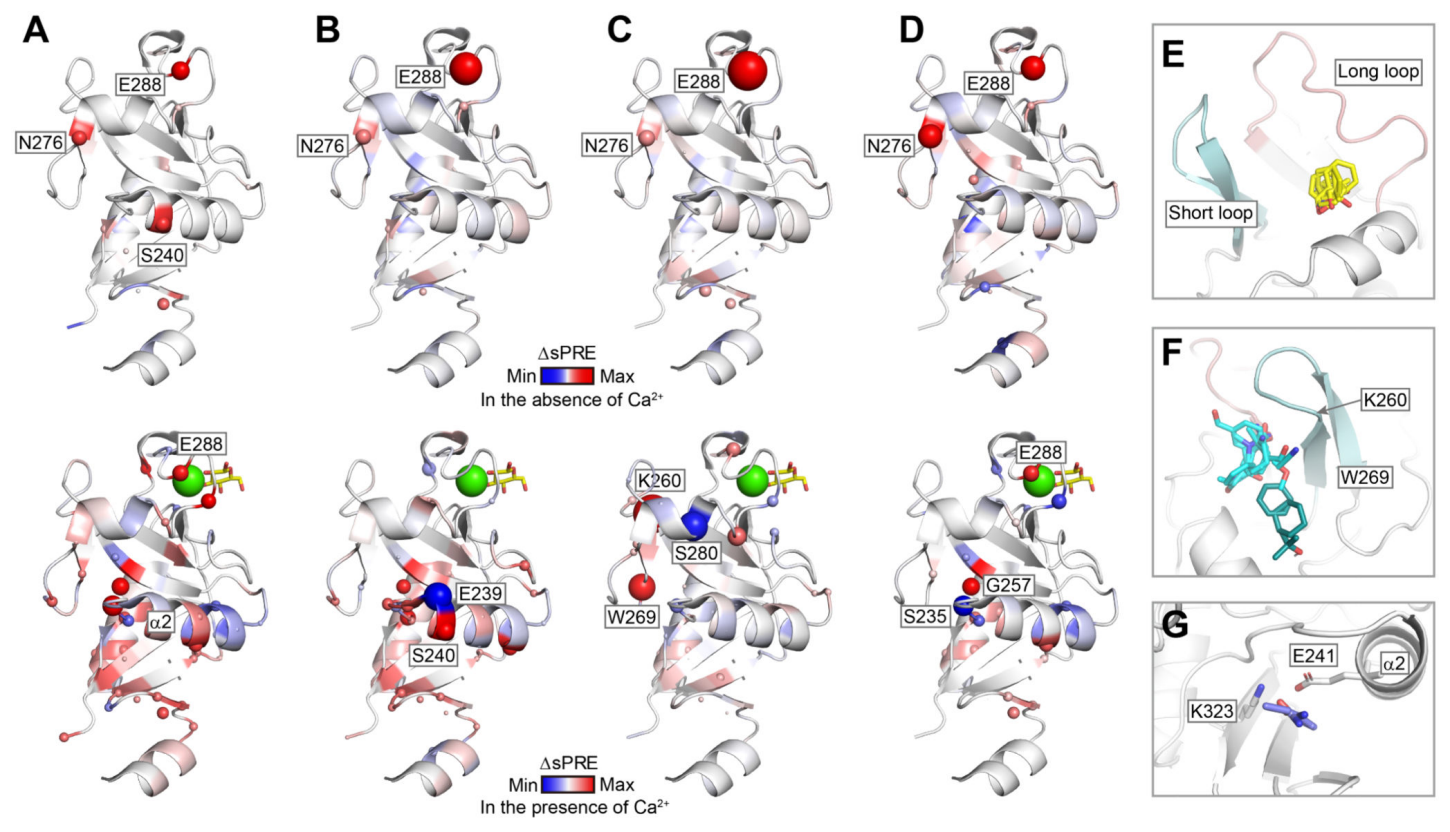
Potential secondary binding sites on murine langerin. (A−D) Solvent paramagnetic relaxation enhancement (sPRE) data of inhibitors **1−4** obtained in the absence (top) and presence (bottom) of 5 mM Ca^2+^ was mapped onto murine langerin CRD (PDB ID: 5M62). The receptor is presented as a cartoon, with ΔsPRE values mapped onto each structure. The color gradient represents the range of ΔsPRE values, scaled according to the maximum and minimum values in the data set. Sphere size of the spheres is proportional to the magnitude of the absolute ΔsPRE value for the affected residue backbone amide. The carbohydrate binding site is marked by glucose in yellow sticks, and Ca^2+^ in green sphere. (E−G) FTMap predicts hotspots on murine langerin CRD, which is shown as white cartoon (PDB ID: 5M62 was used as the starting point for molecular modeling). The probes (stick representation) populating different hot spots are colored accordingly. (E) Primary hot spot 1 overlaps with the X-ray-identified binding pocket; clusters of probes such as phenol, benzaldehyde, and benzene resemble the thiazolopyrimidinone scaffold. (F) Primary hot spot 2 lies adjacent to the short loop in a larger pocket with multiple binding modes and interactions involving Lys260 and Trp269. (G) Secondary hot spot is located in the lower lobe near Glu241 and Lys323, with representative probes including methanamine, ethanol, and urea, suggesting a potential salt bridge or hydrogen bond with polar ligand moieties.

In the absence of Ca^2+^, compounds **1−4** all displayed a strong positive sPRE effect on Glu288 and Asn276, corroborating the primary inhibitor binding site revealed in X-ray (Figure 6A−D). In the presence of Ca^2+^, inhibitors **1**, **2**, and **4** produced strong effects in the receptor’s lower lobe, with the strongest effects observed near the *α*2 helix, particularly at Glu239, Ser240, Ser235, and Gly257 (Figure 6A,B,D). These results suggest that a previously uncharacterized binding site is located near the *α*2 helix, which may reflect low-affinity or allosteric binding events not captured in the ITC experiments and X-ray structures. Additionally, the long and short loops displayed sPRE effects. Inhibitor **3**, in particular, caused a significant effect on Lys260, corroborating the observations from our X-ray studies. Weaker effects on the long loop were observed for all inhibitors, which might be tied to Ca^2+^ release. Notably, inhibitors **2** and **3** induced stronger sPRE signals than inhibitors **1** and **4**, likely reflecting their higher affinities.

Further evidence supporting the primary and secondary sites for thiazolopyrimidinones comes from FTMap, which scores binding sites based on clustering of organic probes on the surface of the protein.^56^ We found two main hot spots and one secondary hot spot of interest. The two main hot spots have clusters of all solvent probes and involve the residues showing much higher contact frequencies in the computation (Figure S7). One hotspot sits between the long and short loop, overlapping with the binding pocket revealed by X-ray (Figure 6E). The other main hotspot is located next to the short loop in a larger pocket that allows for multiple binding modes of the probes (Figure 6F). Here, interactions can be formed with residues Lys260 and Trp269, which align with the sPRE result of inhibitor **3**. The secondary hot spot was found in the lower lobe of the receptor near Glu241 and Lys323, which may form salt bridges or hydrogen bonds with charged ligands (Figure 6G). Carboxylic acid or amine-bearing inhibitors **1−4** could potentially be accommodated in this pocket, which is supported by the sPRE effects near the *α*2 helix.

Taken together, these observations validated the binding site pointed out by X-ray and suggested potential secondary binding sites for inhibitors **1−4**.

## Conclusions

In this study, we have uncovered a previously unrecognized mode of inhibition of murine langerin by thiazolopyrimidinone fragments. Contrary to earlier observations with more evolved fragments, the smallest fragments in this series reported here (**1−4**) induce Ca^2+^ release from the receptor, as demonstrated by ^43^Ca NMR spectroscopy. This is remarkable, as the Ca^2+^ affinity of murine langerin was found to be significantly higher compared to its human homolog.^33^ Consistent with the observation of Ca^2+^ release, ITC data strongly suggest a competitive relationship between these inhibitors and Ca^2+^, and yielded two-digit micromolar affinities for low molecular weight compounds **2** and **3**. In contrast, compounds **1** and **4** showed a much weaker affinity, which suggests that the methyl groups are crucial for determining the binding profile in future fragment extension efforts. ^19^F NMR further validated this competitive relationship, as indicated by inhibition of carbohydrate binding. X-ray crystallography revealed the binding site of inhibitor **3** and the long loop sensitivity toward Ca^2+^. The space-restrained pocket further highlights the importance of the complementarity to the binding site and provides guidance for further fragment growth based on **1−4**. The sPRE NMR data and FTMap additionally suggested a remote secondary binding site. While this secondary site likely exhibits much lower affinity and was therefore not visible in the cocrystal structure, it may be relevant for larger compounds.

The results of our study contribute to the understanding of inhibiting carbohydrate binding via inhibitor-Ca^2+^ competition and localize the binding site of the studied thiazolopyrimidinone derivatives beneath the long loop of murine langerin. Given the conserved sequence and structural features of this loop among several C-type lectins, including human langerin, DC-SIGN, mannose-binding lectin, and BDCA-2, this newly identified site and inhibitory mechanism may offer a conceptual framework for targeting a broader panel of related receptors by the described scaffolds. Our findings emphasize the necessity for structural optimization to enhance inhibitor affinity to establish more stable interactions with a specific site. Although fragment-growing strategies may be limited by the constrained pocket beneath the long loop, similarly sized compounds remain promising. In addition, the identification of potential secondary sites expands the design landscape for larger compounds. It is of interest to explore whether these secondary binding sites trigger the allosteric switch or offer a Ca^2+^-independent binding mode.

Overall, this study presents a robust, multimethod validation of the Ca^2+^ competitive inhibition of murine langerin by thiazolopyrimidinone fragments. The results highlight the long loop as a potential structural switch and offer a design strategy of future langerin and other CTL inhibitors.

## Experimental Section

### Protein Expression and Purification

Murine langerin CRD (amino acids: 191−331) was cloned from a codon-optimized langerin gene for bacterial expression (GenScript, Piscataway, NJ, USA) into a pET-28a vector carrying an N-terminal His-tag with a TEV cleavage site and a T7 promoter. All constructs were expressed in *E. coli* BL21 (ThermoFisher Scientific, Waltham, MA, USA) in LB medium or in isotope-labeled M9 medium at 37 °C. Protein expression was induced by adding 1 mM IPTG at an OD_600_ between 0.7−1.1. Cells were grown for 4 h before harvesting by centrifugation at 4,000 g for 30 min. Bacteria were lysed in lysis buffer (50 mM Tris/HCl pH 7.5, 10 mM MgCl_2_, 1% TritonX-100) with 1 mg/mL lysozyme (Sigma-Aldrich, St. Louis, MO, USA) and 100 *μ*g/mL deoxyribonuclease I from bovine pancreas (Sigma-Aldrich, St. Louis, MO, USA) for 1 h by sonication. The program was set with an on time of 10 s, an off time of 50 s, a total on time of 10 min, and an amplitude of 55%. Inclusion bodies were harvested by centrifugation at 16,000*g* for 30 min and subsequently washed twice with lysis buffer and Milli-Q water. Washed inclusion bodies were solubilized in denaturation buffer (6 M guanidinium hydrochloride in 100 mM Tris/HCl, pH 8) for 1 h at 37°C. After centrifugation (16,000*g*, 60 min, 4 °C), solubilized inclusion bodies were rapidly diluted into refolding buffer (0.8 M L-arginine in 50 mM Tris/HCl, pH 7.6, 20 mM NaCl, 0.8 mM KCl) and stirred overnight at 4 °C. The protein solution was then dialyzed overnight at 4 °C against 50 mM Tris/HCl, pH 7.8, 150 mM NaCl. After another dialysis step, precipitated protein was removed by centrifugation (16000*g*, 30 min, 4 °C) and the langerin CRD was purified using Ni^2+^-NTA affinity chromatography according to manufacturer’s instructions (Cytiva, HisTrap HP column). Purified langerin CRD was dialyzed against 25 mM MES/NaOH, pH 6.0, 40 mM NaCl, supplemented with 5 mM CaCl_2_ overnight at 4 °C. Langerin CRD samples were concentrated using centrifugal filtration, and the concentration was quantified via UV spectroscopy (with A_280_, 0.1% = 2.728). Sample purity was analyzed via SDS-PAGE. The protein solution was aliquoted, snap frozen in liquid N_2_, and stored at −80 °C until further usage. Langerin CRD was dialyzed against 25 mM MES/NaOH, 40 mM NaCl with 10 mM EDTA, and subsequently 25 mM MES/NaOH, 40 mM NaCl before applying to the assays that require Ca^2+^-depleted conditions.

### ^43^Ca NMR

^43^Ca NMR samples contain 50 *μ*M langerin CRD in 25 mM MES/NaOH and 40 mM NaCl, pH 6.0, with 2.5 mM ^43^CaCl_2_ (elemental calcium enriched to 57.9%, prepared from CaCO_3_ by hydrolysis with conc. HCl_aq_ followed by drying under vacuum; repeated three times), 400 *μ*M compound, and 10% D_2_O. The negative controls contain the same components except for the protein. The positive control has 5 mM EDTA for fully chelating 2.5 mM Ca^2+^ in 25 mM MES/NaOH and 40 mM NaCl. A spectrum width between 50 and −50 ppm was chosen. NMR spectra were measured at 25 °C on a 500 MHz Bruker NEO spectrometer equipped with a BBO probe. The carrier frequency was set to 0 ppm, and spectra were recorded with a recovery delay of 5 and 1024 scans. All experiments were carried out with constant concentrations, volumes, receiver gains, and numbers of averaged FIDs.

### ITC Measurements

Isothermal titration calorimetry experiments were performed using a PEAQ-ITC from Malvern or a Nano ITC from TA Instruments at 278.15 K. Langerin CRD in 25 mM MES/NaOH pH = 6.0 with 40 mM NaCl was injected in the sample cell (total volume 170−300 *μ*L, receptor concentration 80−100 *μ*M) of the device. The titrant was dissolved in the same buffer as the protein and added in 20 steps of 2.5 *μ*L (first injection, 1.2 *μ*L; total volume, 50 *μ*L) while stirring at 350 rpm. The differential heat of each injection was measured and plotted against the molar ratio. All thermograms were integrated using NITPIC, the titration curves were fitted using SEDPHAT with error estimates using the confidence interval, and all figures were made in GUSSI.

For Ca^2+^ titration, calcium chloride solutions were prepared in 25 mM MES/NaOH, 40 mM NaCl in the syringe. Langerin CRD was dialyzed against 25 mM MES/NaOH, 40 mM NaCl with 10 mM EDTA, and subsequently 25 mM MES/NaOH, 40 mM NaCl, to remove Ca^2+^ before loading into the sample cell. The thermogram of Ca^2+^ titration was fitted with the One-Site (independent) model. In the Ca^2+^ present titration set up, the protein was in 25 mM MES/NaOH, 40 mM NaCl with 5 mM calcium chloride, and 5% DMSO in the sample cell. The ligand concentration was set to 0.1−2 mM, depending on the solubility, and dissolved in the same buffer as the protein. The raw data were fitted using either the One-Site (independent) model or the Competitive Binding model.^50^ Affinity and enthalpy data of Ca^2+^ binding to langerin were extracted from the Ca^2+^ titration and applied in the Competitive Binding model subsequently. In the Ca^2+^ absent titration set up: ligand concentration was set to 0.1−2 mM, receptor concentration was set to 80−100 *μ*M, both dissolved in 25 mM MES/NaOH, 40 mM NaCl with 5%DMSO. The thermogram was fitted with the one-site (independent) model.

### Ligand Efficiency

Ligand efficiency is calculated based on eq 1:

(1) $$\;\text{ligand}\;\text{efficiency}\;=\frac{\;\text{Gibbs}\;\text{free}\;\text{energy}\;\left(\text{kcal}\cdot {\text{mol}}^{-1}\right)}{\;\text{Number}\;\text{of}\;\text{nonhydrogen}\;\text{atoms}\;}$$

### Inhibition Experiments via ^19^F NMR. ^19^

F-NMR and ^19^F-R_2_-filtered NMR experiments were conducted on a Bruker UltraShield 500 MHz spectrometer at 298 K. Spectra were processed in MestReNova 12.0, and data analysis was performed with GraphPad Prism version 10.2.0 for Windows (GraphPad Software, Boston, Massachusetts, USA, www.graphpad.com). Relaxation rates *R*_2,obs_ were determined with the CPMG pulse sequence, eq 2 shown below. *T* represents the relaxation time, and *I*_0_ is the integral at a *T* value of 0 s. The relaxation delay *d*_1_ was set to 2.0 s, the acquisition time *t*_acq_ was set to 0.87 s, and the frequency of 180° pulses *ν*_CPMG_ was set to 500 Hz.

(2) $$I=I_{0}e^{-R_{2,\text{obs}}T}$$

The *K*_*D*_ value of the reporter was determined in a titration experiment at five concentrations [*L*]_T_ with 50 *μ*M protein. Samples were prepared via serial dilution. The *K*_D_ and the *R*_2,b_ value of the reporter molecule were derived from eq 3 by detection of the ^19^F NMR relaxation rate *R*_2,obs_ in a two-parameter fit. *R*_2,b_ represents the relaxation rate in the bound state of the ligand, and *p*_b_ is the bound fraction of the ligand, while [*P*]_T_ represents the concentration of the receptor. The relaxation rate of the free ligand *R*_2,f_ was measured at 0.1 mM in the absence of the receptor. Standard errors were derived directly from three independent experiments.

(3) $$\begin{array}{l} R_{\text{2,obs}}=R_{2,\text{f}}+\left(R_{2,\text{b}}-R_{2,\text{f}}\right)p_{\text{b}}\\ p_{\text{b}}=\\ \frac{{\left[P\right]}_{\text{T}}+{\left[L\right]}_{\text{T}}+K_{D}-\sqrt{{\left({\left[P\right]}_{\text{T}}+{\left[L\right]}_{\text{T}}+K_{D}\right)}^{2}-4{\left[P\right]}_{\text{T}}{\left[L\right]}_{\text{T}}}}{2{\left[L\right]}_{\text{T}}} \end{array}$$

Due to the complex equilibrium and competition effects present in the competitive binding experiments, EC_50_ values were utilized to quantify the inhibition degree. Samples were prepared via serial dilution. Equation 4 served to derive EC_50_ values from *R*_2,obs_ values. Standard errors were derived directly from three independent experiments. *R*_2,max_ represents the relaxation rate of 50 *μ*M reporter with 50 *μ*M protein, 10 mM Ca^2+^, and relative inhibitor concentration. [Ca^2+^] represents the total concentration of Ca^2+^ in the sample.

(4) $$R_{\text{2,obs}}=R_{2,\text{f}}+\left[{\text{Ca}}^{2+}\right]\left(\frac{R_{2,\text{max}}-R_{2,\text{f}}}{{\text{EC}}_{50}+\left[{\text{Ca}}^{2+}\right]}\right)$$

Titration experiments with glycomimetic were conducted in 10% D_2_O, 25 mM Tris, and 40 mM NaCl, at pH 6.0. TFA served as an internal reference at a concentration of 100 *μ*M.

### sPRE NMR

sPRE experiments were performed on a 500 MHz Bruker Ascend or Ultrashield (Bruker, Billerica, MA, USA) equipped with a CryoProbe Prodigy. Paramagnetic NMR samples contain 100 *μ*M langerin CRD in 25 mM MES, 40 mM NaCl, 5 mM CaCl_2_, 15 mM TEMPOL, and 30 mM ascorbate in the control experiment. Inhibitor concentration was set to 400 *μ*M. The ^1^H−^15^N HMQC was performed with a sequence of sfhmqcf3gpph (NS = 48, TD1 = 1024, TD2 = 256). NMR data were processed and analyzed by CCPN 3.1.1.^57,58^ The ΔsPRE was calculated based on eq 5 described below:

(5) $$\begin{array}{c} \Delta sPRE=sPRE_{\text{protein}+\text{ligand}}-sPRE_{\text{protein}}\\ =\frac{I_{\text{protein}+\text{TEMPOL}+\text{ligand}}}{I_{\text{protein}+\text{ligand}+\text{TEMPOL}+\text{Ascorbate}}}\\ -\frac{I_{\text{protein}+\text{TEMPOL}}}{I_{\text{protein}+\text{TEMPOL}+\text{Ascorbate}}} \end{array}$$

where *I*_protein+TEMPOL_ is the signal intensity when adding TEMPOL, *I*_protein+TEMPOL+Ascorbate_ is the signal intensity when the control sample is quenched with ascorbate, *I*_protein+TEMPOL+ligand_ is the signal intensity with the presence of the ligand and TEMPOL, and *I*_protein+TEMPOL+ligand+ascorbate_ is the signal intensity with the presence of the ligand, TEMPOL, and ascorbate. Signals that have an S/N lower than 10 are excluded from the data analysis. Error bars are plotted according to the S/N.

### Chemistry

All chemicals were purchased from commercial suppliers: Sigma-Aldrich, Alfa Aesar, TCI Chemicals, ChemDiv, and Enamine. Compounds **1−3** and **5** were purchased from BLDPharm. They were used as received unless otherwise specified. NMR spectra were recorded at either 295 K (300 MHz) or 300 K (600 MHz) at either Bruker AV 300 (300 MHz, 75 MHz) or Bruker AV 600 (600 MHz, 151 MHz) spectrometers. Chemical shifts are reported in ppm (*δ*) referenced to residual solvent peak, such as DMSO (^1^H NMR: 2.50 ppm) and CHCl_3_ (^1^H NMR: 7.26 ppm). LC/MS analysis was performed on an Agilent LC/MS 1260 analytical HPLC instrument with DAD coupled to an Agilent 6120 single quadrupole mass spectrometer (ESI-SQ) equipped with a Thermo Fisher Scientific Accucore C18 column, 2.1 mm × 30 mm, 2.6 *μ*m. Method: ESI+, flux: 0.8 mL/min, 5−95% CH_3_CN in H_2_O + 0.1% FA, total runtime: 2.5 min. High-resolution mass spectra were recorded on an Agilent 6220A accurate-mass time-of-flight mass spectrometer (ESI-TOF) with an Agilent 1200 HPLC/DAD front-end. The HPLC was equipped with an Agilent Poroshell 120, C18 column, 2.1 mm × 100 mm, 1.8 *μ*m. Method: ESI+, flux: 0.6 mL/min, 5−99% CH_3_CN in H_2_O + 0.1% FA, total runtime: 4.5 min. Purity and characterization of the compound were established by a combination of LC-MS, LC-HRMS, and NMR analytical techniques. All tested compounds were found to be >95% pure by LC-MS and HRMS analysis.

### *N*-(2-Aminoethyl)-4-oxo-4*H*-pyrido[1,2-*a*]pyrimidine-3-carboxamide (4)

To a solution of 4-oxo-4*H*-pyrido[1,2-*a*]pyrimidine-3-carboxylic acid (57 mg, 0.30 mmol, 1.00 equiv) in DCM (5 mL) was added HATU (148 mg, 0.39 mmol, 1.30 equiv) and DIPEA (157 *μ*L, 0.90 mmol, 3.00 equiv). The mixture was stirred at room temperature for 15 min, and *N*-boc-ethylenediamine (53 mg, 0.33 mmol, 1.10 equiv) was added and stirred for another 2 h. After completion, the reaction mixture was diluted with water (30 mL) and was extracted 3 times with DCM (15 mL). The combined organic phases were dried over MgSO_4_ and concentrated under reduced pressure. The obtained Boc-protected crude was deprotected by stirring in 20% TFA (V/V) in DCM (2 mL) for 2 h at room temperature. The solution was coevaporated with toluene twice to give the crude primary amine TFA salt. The crude product was purified by reversed-phase preparative HPLC (5% to 99% acetonitrile with 0.1% TFA) to yield the desired product 4 as an amorphous white solid (49 mg, 47%). LC-MS (ESI) (*m/z*) [M + H]^+^= 233.1; HRMS (ESI) (*m/z*): [M + H]^+^ calcd for C_11_H_13_N_4_O_2_ [M + H]^+^, 233.1033; found 233.1015. ^1^H NMR (300 MHz, DMSO-*d*_6_) *δ* 9.22 (dd, *J* = 7.4, 1.5 Hz, 1H), 9.16 (t, *J* = 6.0 Hz, 1H), 9.06 (s, 1H), 8.23 (ddd, *J* = 8.6, 6.8, 1.5 Hz, 1H), 7.97−7.90 (m, 1H), 7.86 (s, 2H), 7.64 (td, *J* = 7.0, 1.4 Hz, 1H), 3.61 (q, *J* = 6.1 Hz, 2H), 3.03 (q, *J* = 6.4 Hz, 2H) ppm. ^13^C NMR (75 MHz, DMSO-*d*_6_) *δ*: 164.69, 157.57, 157.46, 152.66, 140.68, 128.72, 126.95, 119.17, 106.26, 37.26 ppm.

## Supplementary Material

SI

Supporting Information

## Figures and Tables

**Table 1:** Comparison of the ITC-Derived Affinities under Different Conditions and Fitting Models

ID	Ca^2+^ present		Ca^2+^ absent
	*K*_D_ [single-site model] (A + B↔AB)	*K*_D_ [competitive binding model] (A + B + C ↔AB + C ↔AC + B)	*K*_D_ [single-site model] (A + B ↔AB)
**1**	N.D.	N.D.	1.1 ± 0.3 mM
**2**	400 ± 70 *μ*M	81 ± 12 *μ*M	19 ± 2 *μ*M
**3**	150 ± 40 *μ*M	17 ± 4 *μ*M	53 ± 8 *μ*M
**4**	8.5 ± 2.0 mM	480 ± 70 *μ*M	200 ± 90 *μ*M

The titrations conducted in the presence of Ca^2+^ were analyzed using both a single-site model and a competitive binding model,^50^ while titrations performed in the absence of Ca^2+^ were fitted to a single-site model. The lower *c*-values and complex interaction resulted in hyperbolic curves in Ca^2+^-present conditions. All data were analyzed using SEDPHAT. Affinities are reported as mean ± standard error from respective fitting models. If binding could not be confidently determined in these experiments, the results are denoted as not detected (N.D.).

## Abbreviations Used

Ca^2+^: calcium ion
CRD: carbohydrate recognition domain
CTL: C-type lectin
HMQC: heteronuclear multiple quantum coherence spectroscopy
ITC: isothermal titration calorimetry
LE: ligand efficiency
NMR: nuclear magnetic resonance
RMSD: root mean square deviation
SPR: surface plasmon resonance
sPRE: solvent paramagnetic relaxation enhancement
