# Supplementary material for: Calcium Competitive Inhibition of Langerin by Thiazolopyrimidinones
Source: J Med Chem. 2025 Nov 19;68(23):24924–34. doi: 10.1021/acs.jmedchem.5c01756 (PMC7618453; doi:10.1021/acs.jmedchem.5c01756)
Supplement: Supplementary file 3 [file jm5c01756_si_003.pdf]

Supporting Information

Calcium Competitive Inhibition of Langerin by Thiazolopyrimidinones

Yunzhan Ning,[1,2] Nina-Louisa Efrém,[4] Machoud Amoussa,[4] Ertan Turhan,[5] Dazhong Zheng,[6,7] Jonathan Lefèbre,[1,2] Max Ruwolt,[8] Ursula Neu,[8] Maurice Besch,[1,2] Bernhard Loll,[8] Dennis Kurzbach,[5] Jesko Köhnke,[6,7] Marc Nazaré,[4] Christoph Rademacher[1,3]*

[1] Department of Pharmaceutical Sciences, University of Vienna, Josef-Holaubek-Platz 2, 1090 Vienna, Austria;

[2] Vienna Doctoral School of Pharmaceutical, Nutritional and Sport Sciences, University of Vienna, Josef-Holaubek-Platz 2, 1090 Vienna, Austria;

[3] Max Perutz Labs, Vienna Biocenter Campus (VBC), Dr.-Bohr-Gasse 9, 1030, Vienna, Austria;

[4] Leibniz-Forschungsinstitut für Molekulare Pharmakologie (FMP), Department of Chemical Biology, Campus Berlin-Buch, Robert-Rössle-Str. 10, 13125 Berlin, Germany;

[5] Institute of Biological Chemistry, Faculty of Chemistry, University of Vienna, Vienna 1090, Austria; University of Vienna, Währinger Straße 38, 1090 Vienna, Austria.

[6] Institute of Food Chemistry, Leibniz University Hannover, Callinstraße 5, 30167 Hannover, Germany;

[7] School of Chemistry, University of Glasgow, University Avenue, Glasgow, G12 8QQ, United Kingdom;

[8] Institute of Chemistry and Biochemistry, Laboratory of Structural Biochemistry, Freie Universität Berlin, Takustr. 6, 14195 Berlin, Germany.

*To whom correspondence should be addressed christoph.rademacher@univie.ac.at

Table of Contents

Table of Contents....................S2
Solubility test....................S3
  Figure S1. Solubility assessment of compound 3 and 5 using ^{1}H NMR....................S3
^{43}Ca NMR....................S4
  Figure S2. ^{43}Ca NMR assay under pH 7.8 suggesting enhanced langerin-....................S4
  Figure S3 Superimposed ^{43}Ca NMR spectra scaled to the same signal amplitude. S5
  Table S1 Parameters of the signals in ^{43}Ca NMR assay....................S5
Isothermal Titration Calorimetry (ITC)....................S6
  Figure S4. ITC thermogram obtained in the presence of Ca^{2+} fitted with one-site binding model. S6
X-ray Crystallographic Procedures S7
  Crystallization S7
  Structure determination and refinement S7
  Table S2. Crystallographic data collection and model refinement statistics....................S8
  Figure S5 Polder map for compound 3 bound to murine langerin....................S9
Molecular Docking General remarks....................S9
  Figure S6. Ligand 3 docking result. S10
  Figure S7. Noncovalent and hydrogen bond contacts fingerprint of murine langerin. S10
Compound spectra....................S11
  Figure S8. $^{1}H/^{13}C$-NMR for Compound 4 ....S11
  Figure S9. HPLC spectrum of compound 4....................S12
  Table S3. SMILES of compounds used in the study S12
References....................S13

Solubility test

The compounds were dissolved in 100 mM DMSO and titrated into the same buffer solution where the protein was stored for different essays. DSS was used as the reference with a concentration of 100 μM. The integration and chemical shift of the ligand signals were monitored as indicators of potential aggregation. The solubility of compounds **1**, **2**, **3** were reported elsewhere.[1]

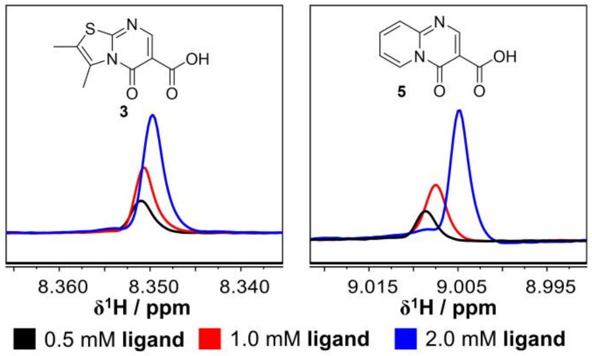

Figure S1. Solubility assessment of compound 3 and 5 using ^{1}H NMR. Compound **3** displayed no observable chemical shifts within 1 mM and the integration is consistent with the expected ratio relative to DSS, indicating its solubility at 1 mM in aqueous buffer. In contrast, compound **5** exhibited chemical shifts and integration values below the expected DSS ratio from 1 mM, suggesting partial solubility.

^{43}Ca NMR

Data processing and visualization were performed using TopSpin 4.4.0 (Bruker BioSpin GmbH, Rheinstetten, Germany) and MestReNova 10.0 (Mestrelab Research, Santiago de Compostela, Spain). Spectra were processed using an exponential window function with 1.00 Hz, zero-filled to a final size of 32,768 points (from an original FID size of 3,424), and baseline-corrected using spline. Parameter extraction was performed on data fitted without a window function to ensure an unbiased representation of the spectral features.

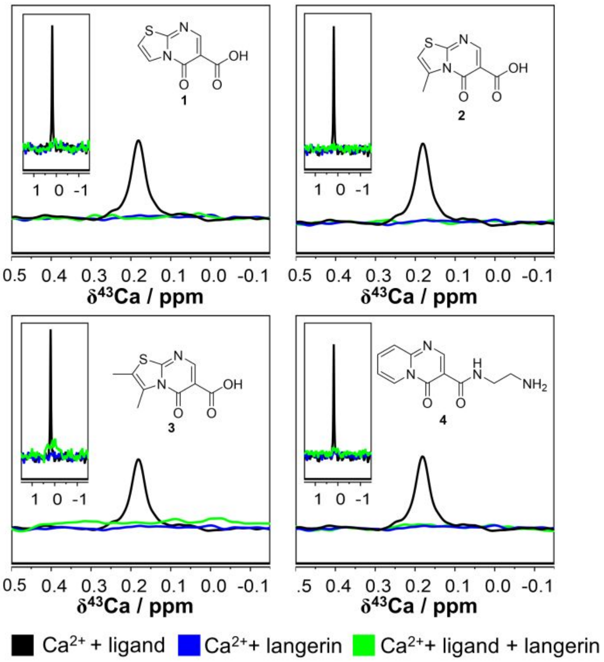

Figure S2. ^{43}Ca NMR assay under pH 7.8 suggesting enhanced langerin-Ca^{2+} **complex formation.** ^{43}Ca NMR spectra of 2.5 mM $^{43}CaCl_2$ in 25 mM Tris/HCl, 150 mM NaCl, pH 7.8, were recorded in the absence (black) and presence (blue) of 50 µM langerin CRD. The spectra in green represent the addition of 400 µM ligands (**1**-**4**) to the langerin-Ca^{2+} complex. In the expanded view with broader spectra width, signals with larger linewidth and reduced intensity were observed compared with the signals resulted from under pH 6.0 (**Figure 1C**). The significant reduction in signal intensity upon langerin addition suggests stronger langerin-Ca^{2+} complex formation at this pH.

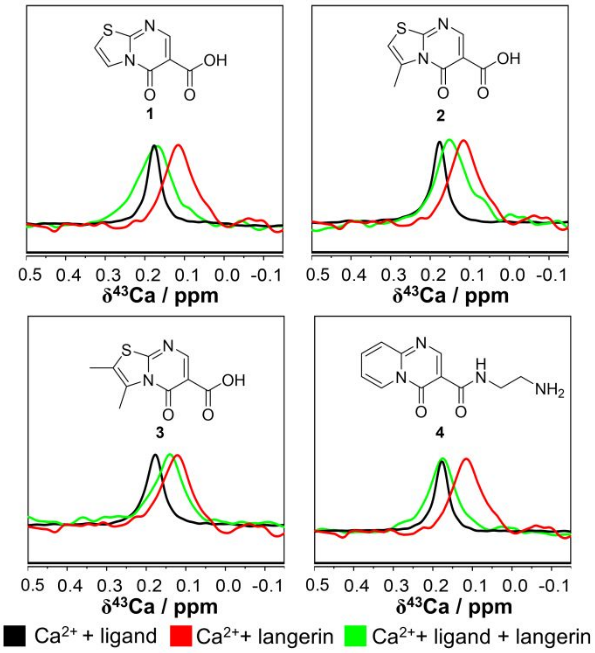

Figure S3 Superimposed ^{43}Ca NMR spectra scaled to the same signal amplitude. The chemical shift perturbation effect of 400 µM inhibitors (**1**-**4**) on Ca^{2+} binding to langerin was analyzed in the presence of 2.5 mM $^{43}CaCl_2$ (25 mM MES/NaOH, 40 mM NaCl, pH 6.0). Spectra are shown for Ca^{2+} alone with inhibitors (black), Ca^{2+} with langerin and inhibitors (green), and Ca^{2+} with langerin alone (red). Shifts in the ^{43}Ca resonance indicate competitive inhibition of Ca^{2+} binding by the ligands.

Table S1 Parameters of the signals in ^{43}Ca NMR assay.

	**Ca^{2+} (free)**	**Ca^{2+} + langerin pH 6.0 (bond)**	**Ca^{2+} + langerin + 1**	**Ca^{2+} + langerin + 2**	**Ca^{2+} + langerin + 3**	**Ca^{2+} + langerin + 4**
**Chemical shift δ**	0.175	0.116	0.173	0.154	0.139	0.174
**Linewidth Δν**	0.89	1.94	2.80	1.79	1.78	1.64
**Integral (relative to the ligand present free state)**	1	0.147	0.355	0.324	0.344	0.316

Isothermal Titration Calorimetry (ITC)

The raw ITC data were initially processed using the default software provided by respective ITC instrument: MicroCal PEAQ-ITC Analysis Software 1.41 and Nanoanalyze Data Analysis 3.5.0. For MicroCal, the fitting model was set to *One Set of Sites*, with the following parameters: baseline option (time factor 5, points per injection 25), control parameter set to *Fitted Offset*, site value fixed at 1 and other parameters set to vary. For Nanoanalyze, blank titration files were used for area correction, fitting model set to *independent*, binding site *n* fixed at 1, and other parameters allowed to vary.

The raw data from both systems were further processed using NITPIC 2.1.0, baseline type set to *Best Fit*, with pre- and post-injection tail correction applied, integral significance level was adjusted based on the quality of each titration, and the outlier threshold was defined as twice of the standard deviation. The processed data were then analyzed in SEDPHAT 15.2b. For Ca^{2+} titration and ligand titration in the absence of Ca^{2+}, the fitting model was set as *Hetero Association A-B* (Single-site model) with the global parameter log(Ka) and dHAB set as *vary*. For ligand titration in the presence of Ca^{2+}, the fitting model was set as *Competing B and C for A* (Competitive binding model) with the global parameter log(KAC) and dHAC set as the data obtained from Ca^{2+} titration (4.336 and -8.2 kcal/mol), and log(KAB) and dHAB as *vary*. Experimental parameters for titration were defined as *Titrating B into AC*. Final presentation of the fitting results was performed using GUSSI (version 2.1.6).[2]

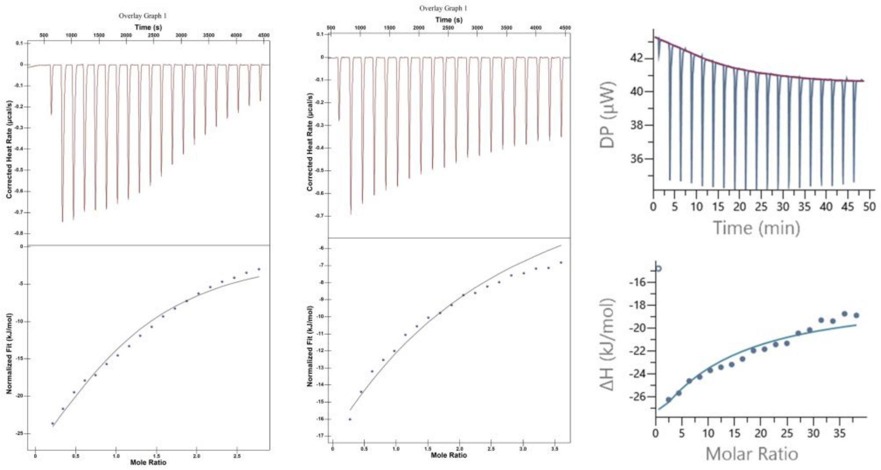

Ligand **1** Ligand **3** Ligand **4**

Figure S4. ITC thermogram obtained in the presence of Ca^{2+} fitted with one-site binding model. The poor convergence of the fit suggests a more complex binding mechanism, highlighting the need for alternative models and additional experiments to fully characterize the thermodynamics of the system.

X-ray Crystallographic Procedures

Crystallization

Heterologously expressed and purified murine langerin CRD was subjected to TEV digestion to remove the His-tag that potentially inhibits crystal formation. The protein (3 mg / mL) was mixed with TEV (1 mg / mL) *v/v* 1 : 10 in a 3,000 kDa MWCO dialysis cassette and incubated overnight under 4 °C. The digested sample was subsequently purified through Ni-NTA affinity chromatography. and the cleavage efficiency was assessed by SDS-PAGE.

Then the protein was concentrated to 7 mg mL^{-1} using centrifugal filters. Crystals were obtained by sitting-drop vapor-diffusion method at 18 °C with a reservoir solution composed of 21% (w/v) polyethylene glycol 3,350 and 200 mM KCl. Drops had a protein:reservoir ratio of 1:4 and were supplemented with 1:10 seed stock. Crystals for the seed stock were grown in 20% polyethylene glycol 3350 and 200 mM NaCl. Before to flash-freezing in liquid nitrogen, crystals were transferred to a cryo-protectant solution containing the reservoir solution and additional 12.5% (v/v) polyethylene glycol 400.

To obtain the complex with **3**, the protein was incubated with 1 mM compound on ice over night before crystallization trials. Crystals were obtained in JCSG Core Suite IV condition C10 (0.1 M Tris pH 8.0, 5% PEG 6,000) using the vapor diffusion method. Crystals were cryoprotected in reservoir solution supplemented with 1mM **3** and 32% glycerol before harvesting and flash-freezing in liquid nitrogen.

Structure determination and refinement

Diffraction data for the apo protein were collected at the beamline 14.2 of the MX Joint Berlin laboratory at synchrotron BESSY (Berlin, Germany), while diffraction data for the complex structure with **3** was collected at ESRF beamline ID30B.

Data were processed with XDS[3] (Table S1). The crystal structure was determined using PHASER[4] molecular replacement with the coordinates of the murine langerin CRD (PDB: 5K8Y[5]) as a search model. Consequently, the structure was refined using the PHENIX[6,7] maximum-likelihood restrained refinement. Water picking and manual model building were carried out with COOT[8]. Model quality was evaluated with MolProbity[9] and the JCSG validation server (JCSG Quality Control Check v3.1). The final structure was visualized in PyMOL (Schrödinger, Inc).

Table S2. Crystallographic data collection and model refinement statistics.

Dataset		
PDB entry	9HYE	9RKO
**Data Collection**		
Wavelength [Å]	0.9184	0.9677
Temperature [K]	100	100
Space group	$P2_1$	$P2_1$
Unit Cell Parameters<br>a, b, c [Å]<br>α, β, γ [°]	 35.1; 81.1; 52.5<br>90.0 105.2 90.0	 35.2; 81.8; 52.2<br>90.0; 104.7; 90.0
Resolution [Å][a]	50.00 - 1.64<br>(1.74 - 1.64)	50.49 – 1.89<br>(1.92 – 1.89)
Reflections [a]<br>Unique [a]<br>Completeness [%][a]<br>Multiplicity [a]	 34,064 (5,380)<br>98.9 (96.6)<br>6.8 (6.5)	 44,473 (7,826)<br>98.7 (99.7)<br>6.7 (6.9)
Data quality [a]<br>Intensity [I/σ(I)] [a]<br>R_{meas} [%][a,b]<br>$CC_{1/2}$ [a,c]<br>Wilson B value [Å^2]	 8.1 (1.0)<br>16.1 (210.9)<br>99.4 (37.0)<br>28.4	 10.4 (2.1)<br>14.5 (98.7)<br>99.5 (70.4)<br>20.4
**Refinement**		
Resolution [Å][a]	50.00 - 1.64<br>(1.70 - 1.64)	50.49 – 1.89<br>(1.93 – 1.89)
Reflections [a]<br>Number<br>Test Set [%]	 33,991 (3,352)<br>5.0	 44,465 (2,841)<br>5.0
R_{work} [%][a]<br>R_{free} [%][a]	15.3 (29.4)<br>21.6 (36.4)	18.0 (26.2)<br>21.1 (29.6)
Asymmetric Unit<br>Protein: Residues, Atoms<br><br>Ligands: Molecules<br><br>Water molecules	 136 (A), 1,203 (A)<br>137 (B), 1,230 (B)<br>3 (Cl^-)<br><br>224	 140(A), 2246 (A)<br>140 (B), 2242 (B)<br>2<br><br>224
Mean Temperature factors [Å^2][b]<br>All Atoms<br>Macromolecules<br>Ligands<br><br>Water molecules	 27.0<br>26.9 (A), 30.5 (B)<br>39.1 (Cl^-)<br><br>35.8	 30.9<br>30.0<br>61.6<br><br>36.0
RMSD from Target Geometry [d]<br>Bond Lengths [Å]<br>Bond Angles [°]	 0.011<br>1.003	 0.003<br>0.63
**Validation Statistics**		
Ramachandran Plot [f]<br>Residues in Allowed Regions [%]<br>Residues in Favored Regions [%]<br>Ramachandran plot *Z*-score [f] (RMSD)<br>whole<br>helix<br>sheet<br>loop<br>MOLPROBITY Clashscore [g]<br>MOLPROBITY score [f]	 1.89<br>98.1<br><br>-1.44 (0.41)<br>-0.80 (0.41)<br>-0.41 (1.10)<br>-1.09 (0.41)<br>1.9<br>1.23	 3.3<br>96.4<br><br>-1.64 (0.43)<br>-0.99 (0.47)<br>-0.50 (0.80)<br>-1.25 (0.43)<br>9.3<br>1.7

[a] data for the highest resolution shell in parenthesis
[b] $R_{meas}(I) = \sum_h [N/(N-1)]^{1/2} \sum_i |I_{ih} - \langle I_h \rangle| / \sum_h \sum_i I_{ih}$, in which $\langle I_h \rangle$ is the mean intensity of symmetry-equivalent reflections h, I_{ih} is the intensity of a particular observation of h and N is the number of redundant observations of reflection h.[10]
[c] $CC_{1/2} = (\langle I^2 \rangle - \langle I \rangle^2) / (\langle I^2 \rangle - \langle I \rangle^2) + \sigma^2_\varepsilon$, in which σ^2_ε is the mean error within a half-dataset.[11]
[d] RMSD – root mean square deviation
[e] calculated with PHENIX
[f] calculated with MOLPROBITY[12]
[g] Clashscore is the number of serious steric overlaps (> 0.4) per 1,000 atoms.[9]

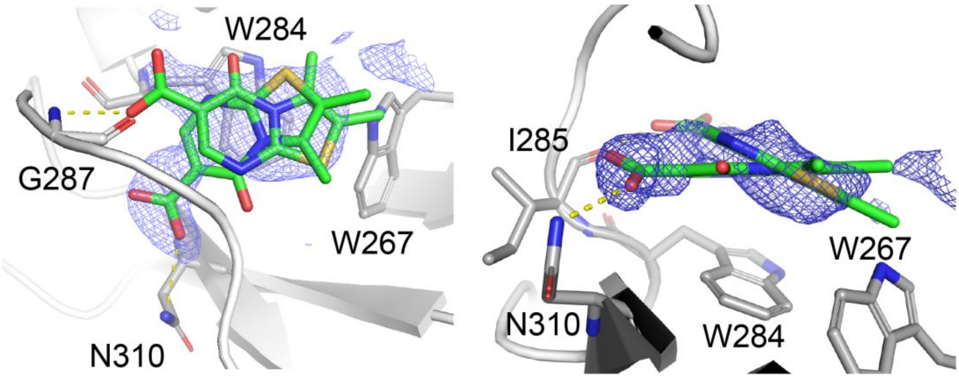

Figure S5 Polder map for compound 3 bound to murine langerin. (contour level: 3.0σ). The electron density beneath the long loop is diffuse, consistent with the presence of multiple binding poses. Occupancy refinement suggests a 1:1 distribution of two orientations: one forming a hydrogen bond with Nα of Gly287, and the other with the side-chain amide of Asn310.

Molecular Docking General remarks.

Molecular docking procedures were performed in Molecular Operating Environment. (MOE 2024.0601 Chemical Computing Group ULC, 910-1010 Sherbrooke St. W., Montreal, QC H3A 2R7, 2024.) The docking method for placement was set to *Alpha Triangle* (London dG, poses 30), and Refinement set as *Rigid Receptor* (GBVI/WSA dG, poses 30). Receptor surfaces were visualized in *Schematic* style. The docking site was defined as the residues surrounding ligand **3** in the corresponding X-ray structure (PDB ID: 9RKO). To explore diverse binding modes, the PH4 option was disabled, allowing the ligand to flip. The site space was restricted by a *Wall Constraint*. Next, protonation states and the hydrogen bond network of the complex were simulated with MOE's Protonate 3D followed by the removal of all solvent molecules. The poses are ranked by the scores from the GBVI/WSA binding free energy.

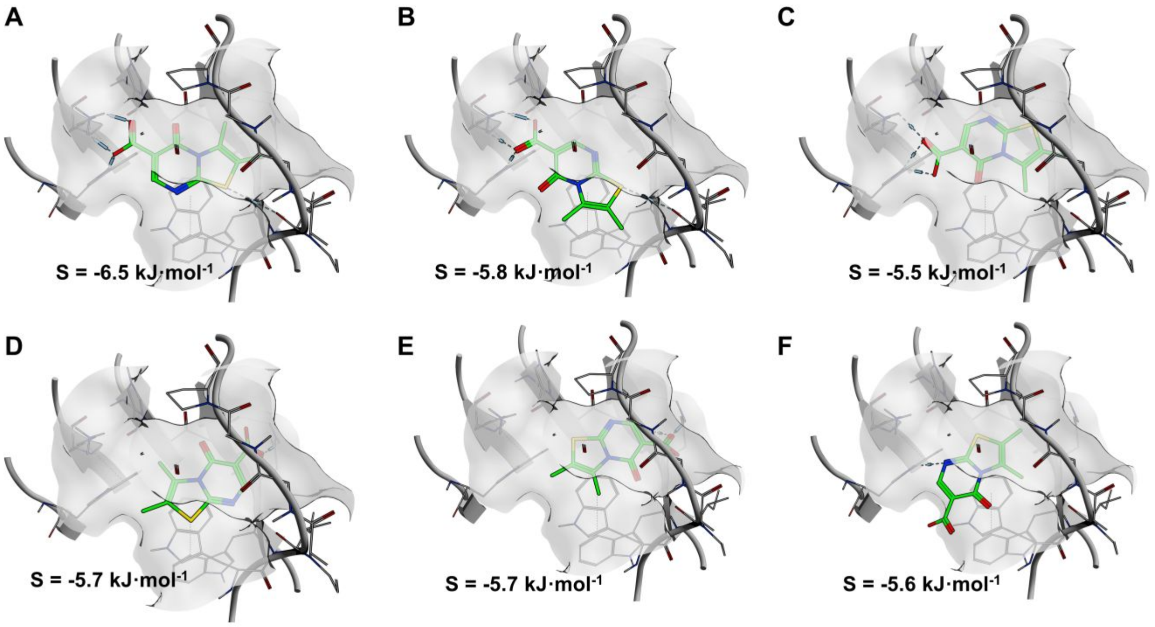

Figure S6. Ligand 3 docking result. (A): binding mode shown in the X-ray structure. (B-F): Predicted binding poses from molecular docking. Key interactions are depicted as blue cylinders, highlighting that binding is primarily driven by steric complementarity and a salt bridge.

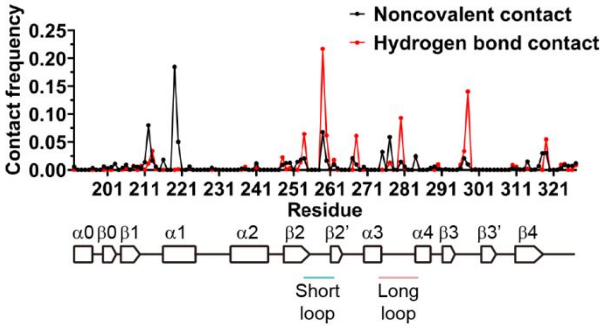

Figure S7. Noncovalent and hydrogen bond contacts fingerprint of murine langerin. The frequency of noncovalent and hydrogen bond contacts between FTMap probes and murine langerin residues is plotted along the sequence. Residues with high contact frequencies support the two primary hot spots described in the main text. Specifically, Trp219 and Thr298 correspond to the hot spot adjacent to the short loop, while Ser280 supports the hot spot located between the long and short loop.

Compound spectra.

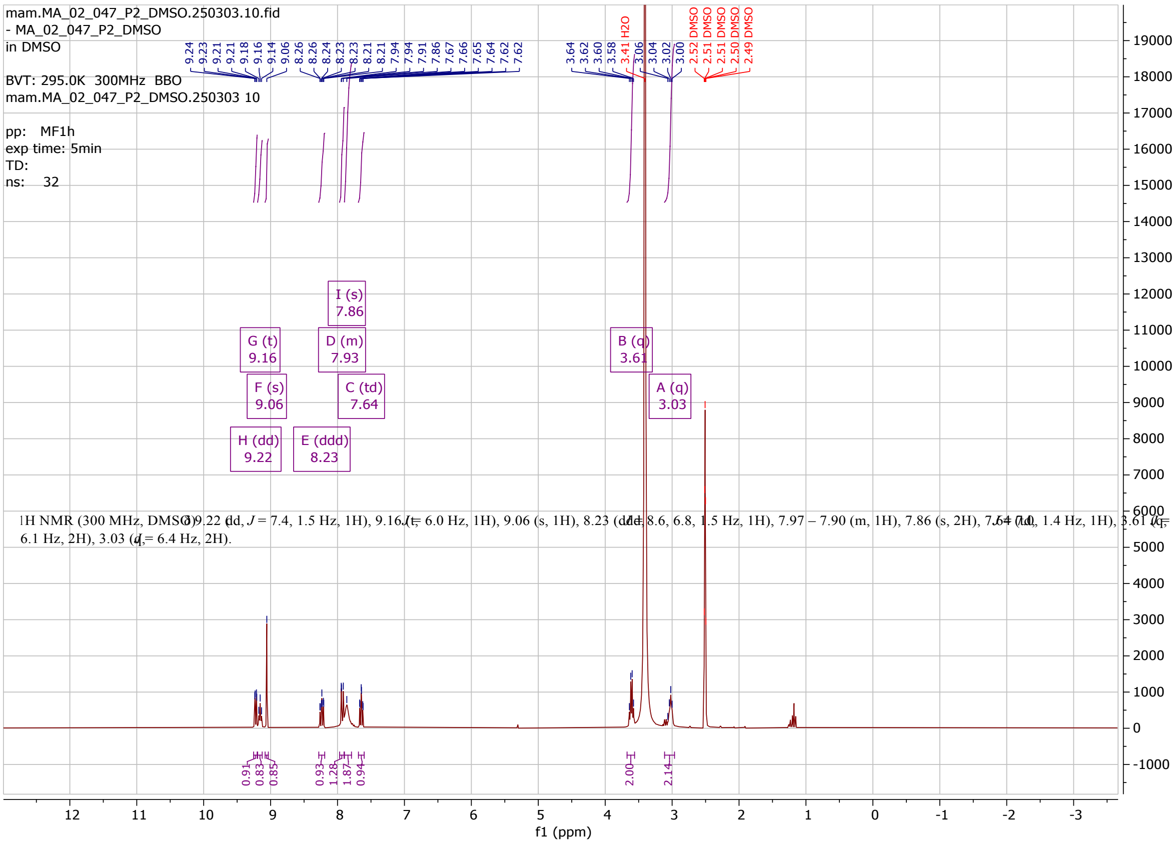

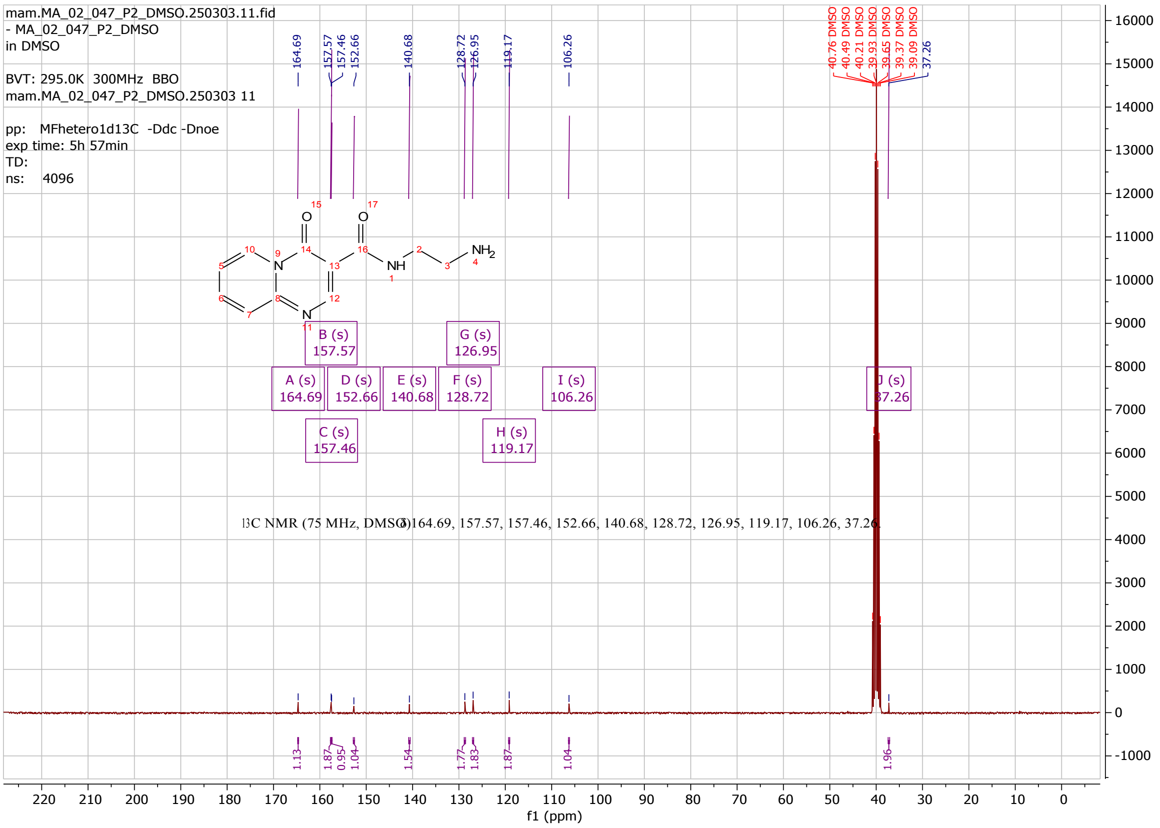

Figure S8. ^{1}H/^{13}C-NMR for Compound 4

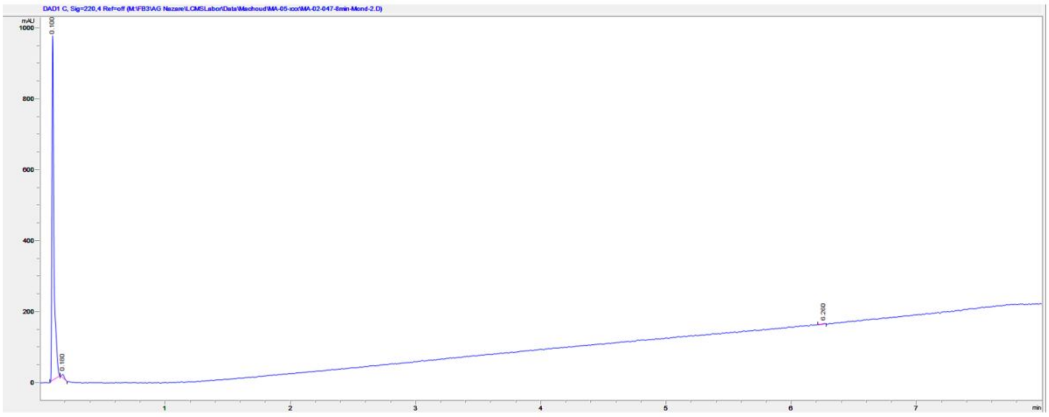

Peak #	Area	Height	Width	Area%	Symmetry
1	1117.9	977.9	0.0168	98.258	0.526

Figure S9. HPLC spectrum of compound 4

Table S3. SMILES of compounds used in the study

Compound	SMILES	K_D (Ca^{2+} present)	K_D (Ca^{2+} absent)
**1**	O=C1N2C=CSC2=NC=C1C(O)=O	-	1.1 ± 0.3 mM
**2**	O=C1N2C(C)=CSC2=NC=C1C(O)=O	81 ± 12 μM	19 ± 2 μM
**3**	O=C1N2C(C)=C(C)SC2=NC=C1C(O)=O	17 ± 4 μM	53 ± 8 μM
**4**	NCCNC(C1=CN=C2C=CC=CN2C1=O)=O	480 ± 70 μM	200 ± 90 μM

References

(1) Aretz, J.; Anumala, U. R.; Fuchsberger, F. F.; Molavi, N.; Ziebart, N.; Zhang, H.; Nazaré, M.; Rademacher, C. Allosteric Inhibition of a Mammalian Lectin. *J. Am. Chem. Soc.* **2018**, *140* (44), 14915–14925. https://doi.org/10.1021/jacs.8b08644.

(2) Brautigam, C. A.; Zhao, H.; Vargas, C.; Keller, S.; Schuck, P. Integration and Global Analysis of Isothermal Titration Calorimetry Data for Studying Macromolecular Interactions. *Nat. Protoc.* **2016**, *11* (5), 882–894. https://doi.org/10.1038/nprot.2016.044.

(3) Kabsch, W. *XDS*. *Acta Crystallogr. D Biol. Crystallogr.* **2010**, *66* (2), 125–132. https://doi.org/10.1107/S0907444909047337.

(4) McCoy, A. J.; Grosse-Kunstleve, R. W.; Adams, P. D.; Winn, M. D.; Storoni, L. C.; Read, R. J. *Phaser* Crystallographic Software. *J. Appl. Crystallogr.* **2007**, *40* (4), 658–674. https://doi.org/10.1107/S0021889807021206.

(5) Hanske, J.; Schulze, J.; Aretz, J.; McBride, R.; Loll, B.; Schmidt, H.; Knirel, Y.; Rabsch, W.; Wahl, M. C.; Paulson, J. C.; Rademacher, C. Bacterial Polysaccharide Specificity of the Pattern Recognition Receptor Langerin Is Highly Species-Dependent. *J. Biol. Chem.* **2017**, *292* (3), 862–871. https://doi.org/10.1074/jbc.M116.751750.

(6) Liebschner, D.; Afonine, P. V.; Baker, M. L.; Bunkóczi, G.; Chen, V. B.; Croll, T. I.; Hintze, B.; Hung, L.-W.; Jain, S.; McCoy, A. J.; Moriarty, N. W.; Oeffner, R. D.; Poon, B. K.; Prisant, M. G.; Read, R. J.; Richardson, J. S.; Richardson, D. C.; Sammito, M. D.; Sobolev, O. V.; Stockwell, D. H.; Terwilliger, T. C.; Urzhumtsev, A. G.; Videau, L. L.; Williams, C. J.; Adams, P. D. Macromolecular Structure Determination Using X-Rays, Neutrons and Electrons: Recent Developments in *Phenix*. *Acta Crystallogr. Sect. Struct. Biol.* **2019**, *75* (10), 861–877. https://doi.org/10.1107/S2059798319011471.

(7) Adams, P. D.; Afonine, P. V.; Bunkóczi, G.; Chen, V. B.; Davis, I. W.; Echols, N.; Headd, J. J.; Hung, L.-W.; Kapral, G. J.; Grosse-Kunstleve, R. W.; McCoy, A. J.; Moriarty, N. W.; Oeffner, R.; Read, R. J.; Richardson, D. C.; Richardson, J. S.; Terwilliger, T. C.; Zwart, P. H. *PHENIX*: A Comprehensive Python-Based System for Macromolecular Structure Solution. *Acta Crystallogr. D Biol. Crystallogr.* **2010**, *66* (2), 213–221. https://doi.org/10.1107/S0907444909052925.

(8) Casañal, A.; Lohkamp, B.; Emsley, P. Current Developments in *Coot* for Macromolecular Model Building of Electron Cryo-microscopy and Crystallographic Data. *Protein Sci.* **2020**, *29* (4), 1055–1064. https://doi.org/10.1002/pro.3791.

(9) Williams, C. J.; Headd, J. J.; Moriarty, N. W.; Prisant, M. G.; Videau, L. L.; Deis, L. N.; Verma, V.; Keedy, D. A.; Hintze, B. J.; Chen, V. B.; Jain, S.; Lewis, S. M.; Arendall, W. B.; Snoeyink, J.; Adams, P. D.; Lovell, S. C.; Richardson, J. S.; Richardson, D. C. MolProbity: More and Better Reference Data for Improved All-atom Structure Validation. *Protein Sci.* **2018**, *27* (1), 293–315. https://doi.org/10.1002/pro.3330.

(10) Diederichs, K.; Karplus, P. A. Improved R-Factors for Diffraction Data Analysis in Macromolecular Crystallography. *Nat. Struct. Biol.* **1997**, *4* (4), 269–275. https://doi.org/10.1038/nsb0497-269.

(11) Karplus, P. A.; Diederichs, K. Linking Crystallographic Model and Data Quality. *Science* **2012**, *336* (6084), 1030–1033. https://doi.org/10.1126/science.1218231.

(12) Williams, C. J.; Headd, J. J.; Moriarty, N. W.; Prisant, M. G.; Videau, L. L.; Deis, L. N.; Verma, V.; Keedy, D. A.; Hintze, B. J.; Chen, V. B.; Jain, S.; Lewis, S. M.; Arendall, W. B.; Snoeyink, J.; Adams, P. D.; Lovell, S. C.; Richardson, J. S.; Richardson, D. C. MolProbity: More and Better Reference Data for Improved All-atom Structure Validation. *Protein Sci.* **2018**, *27* (1), 293–315. https://doi.org/10.1002/pro.3330.
