# Supplementary material for: Calcium Competitive Inhibition of Langerin by Thiazolopyrimidinones
Source: J Med Chem. Author manuscript; Available in PMC 2025 Dec 10. (PMC7618453; doi:10.1021/acs.jmedchem.5c01756)
Supplement: Supplementary file 1 [file EMS211266-supplement-Supporting_Information.pdf]

Supporting Information

The Supporting Information is available free of charge at https://pubs.acs.org/doi/10.1021/acs.jmedchem.5c01756.

Refined 9RKO (PDB)

Refined 9HYE (PDB)

Additional ^{43}Ca NMR spectra; crystallographic data and refinement statistics; docking study; compound spectra and HPLC analysis (PDF)

Molecular formula String file of compounds associated with K_D values (CSV)

..

..

https://doi.org/10.1021/acs.jmedchem.5c01756
J. Med. Chem. XXXX, XXX, XXX−XXX
